# Pho dynamically interacts with Spt5 to facilitate transcriptional switches at the *hsp70* locus

**DOI:** 10.1186/s13072-017-0166-9

**Published:** 2017-12-06

**Authors:** Allwyn Pereira, Renato Paro

**Affiliations:** 1 Department of Biosystems Science and Engineering, ETH Zurich, 4058 Basel, Switzerland; 2 Faculty of Sciences, University of Basel, 4056 Basel, Switzerland

**Keywords:** Heat shock, Pol II pausing, Polycomb proteins, Protein–protein interactions, Transcriptional activators

## Abstract

**Background:**

Numerous target genes of the Polycomb group (PcG) are transiently activated by a stimulus and subsequently repressed. However, mechanisms by which PcG proteins regulate such target genes remain elusive.

**Results:**

We employed the heat shock-responsive *hsp70* locus in *Drosophila* to study the chromatin dynamics of PRC1 and its interplay with known regulators of the locus before, during and after heat shock. We detected mutually exclusive binding patterns for HSF and PRC1 at the *hsp70* locus. We found that Pleiohomeotic (Pho), a DNA-binding PcG member, dynamically interacts with Spt5, an elongation factor. The dynamic interaction switch between Pho and Spt5 is triggered by the recruitment of HSF to chromatin. Mutation in the protein–protein interaction domain (REPO domain) of Pho interferes with the dynamics of its interaction with Spt5. The transcriptional kinetics of the heat shock response is negatively affected by a mutation in the REPO domain of Pho.

**Conclusions:**

We propose that a dynamic interaction switch between PcG proteins and an elongation factor enables stress-inducible genes to efficiently switch between ON/OFF states in the presence/absence of the activating stimulus.

**Electronic supplementary material:**

The online version of this article (10.1186/s13072-017-0166-9) contains supplementary material, which is available to authorized users.

## Background

Polycomb group (PcG) proteins are a highly conserved class of epigenetic regulators that play an essential role during development and adulthood in many organisms [1]. In any given cell type, while PcG proteins maintain stable silencing at a certain subset of genes, other PcG target genes are capable of being activated in the presence of the appropriate stimulus. Thus, by conferring both stability and plasticity upon the transcriptome, PcG proteins bestow competence upon the cells to respond to varied stimuli over their lifetime, in order to maintain cellular homoeostasis [2]. However, the underlying mechanisms that enable PcG-regulated loci to dynamically switch between ON and OFF states remain elusive.

In *Drosophila*, the targeting of PcG proteins to chromatin occurs mainly through the recognition of cis-regulatory elements, known as Polycomb group response elements (PREs), by Pleiohomeotic (Pho) and other DNA-binding proteins [3, 4]. Pho interacts with dSfmbt to form the Pho repressive complex (PhoRC), which acts as a recruitment platform for the Polycomb repressive complex 1 and 2 (PRC1 and PRC2) [5, 6]. Subsequently, PRC1 and PRC2 generate a chromatin structure that is incompatible with gene expression, through their enzymatic and chromatin compaction activities [7, 8]. The transcriptionally silent state is then faithfully inherited through cell division, which involves PRE-dependent mechanisms [9, 10]. Although the mechanisms by which PcG proteins establish and maintain stable silencing at their targets have been subject to intense investigation, little is known about PcG-mediated regulation of target genes with dynamic expression patterns.

In this study, we employed the heat shock-responsive *hsp70* gene in *Drosophila* as a model to study dynamic gene regulation by PcG proteins (Additional file 1: Figure S1a). The *hsp70* locus is an attractive inducible gene model as it is targeted by PRC1 [11]. It is regulated at the level of transcriptional elongation by promoter-proximal RNA polymerase II pausing [12]. At normal growth temperature (25 °C), RNA polymerase II is maintained in the paused state by the DSIF complex (composed of Spt4–Spt5) and the NELF complex [13, 14]. The heat shock stimulus leads to the recruitment of the transcription factor, HSF, to chromatin [15]. This is followed by the recruitment of P-TEFb, which phosphorylates Spt5 and converts it to function as a positive elongation factor [16, 17]. This leads to rapid up-regulation of the *hsp70* locus in response to the heat stress [18].

While the transcriptional response and the chromatin dynamics at the *hsp70* locus have been well characterised in the acute phase of the heat shock response, the effect of the withdrawal of the activating stimulus on the transcription and chromatin dynamics remains poorly understood [18]. By following the chromatin dynamics of PRC1 and Pho during the recovery phase of the heat shock, we sought to dissect the role of these two factors in the re-establishment of silencing at the *hsp70* locus. Pho was previously found to co-localise with RNA polymerase II upon heat shock [19]. Furthermore, Pho interacts with Spt5 [20], and Spt5 is involved in both, promoter-proximal pausing and transcriptional elongation [21]. We thus asked whether the conversion of Spt5 from a pausing factor into an elongation factor upon gene activation and its interaction with Pho could mechanistically explain the spreading of Pho in the body of active genes. Genetic analyses revealed the necessity of Pho gene function in the re-establishment of silencing at the *hsp70* locus in the absence of the activating stimulus [19]. We tested the hypothesis whether Pho could dynamically interact with repressors (PRC1 via dSfmbt) and activators (Spt5) during the heat shock response and the significance of its dynamic protein–protein interactions on transcriptional regulation of the *hsp70* locus.

By combining multiple read-outs, we identified a role for the dynamic protein–protein interaction between Pho and Spt5 in the transcriptional regulation of the *hsp70* locus. Our results highlight how a dynamic interaction switch between PcG proteins and an elongation factor could facilitate PcG-regulated genes, which are responsive to inductive signals, to efficiently switch between ON/OFF states.

## Results

### Antagonistic binding patterns of HSF and PRC1 at the *hsp70* locus

**Fig. 1:**
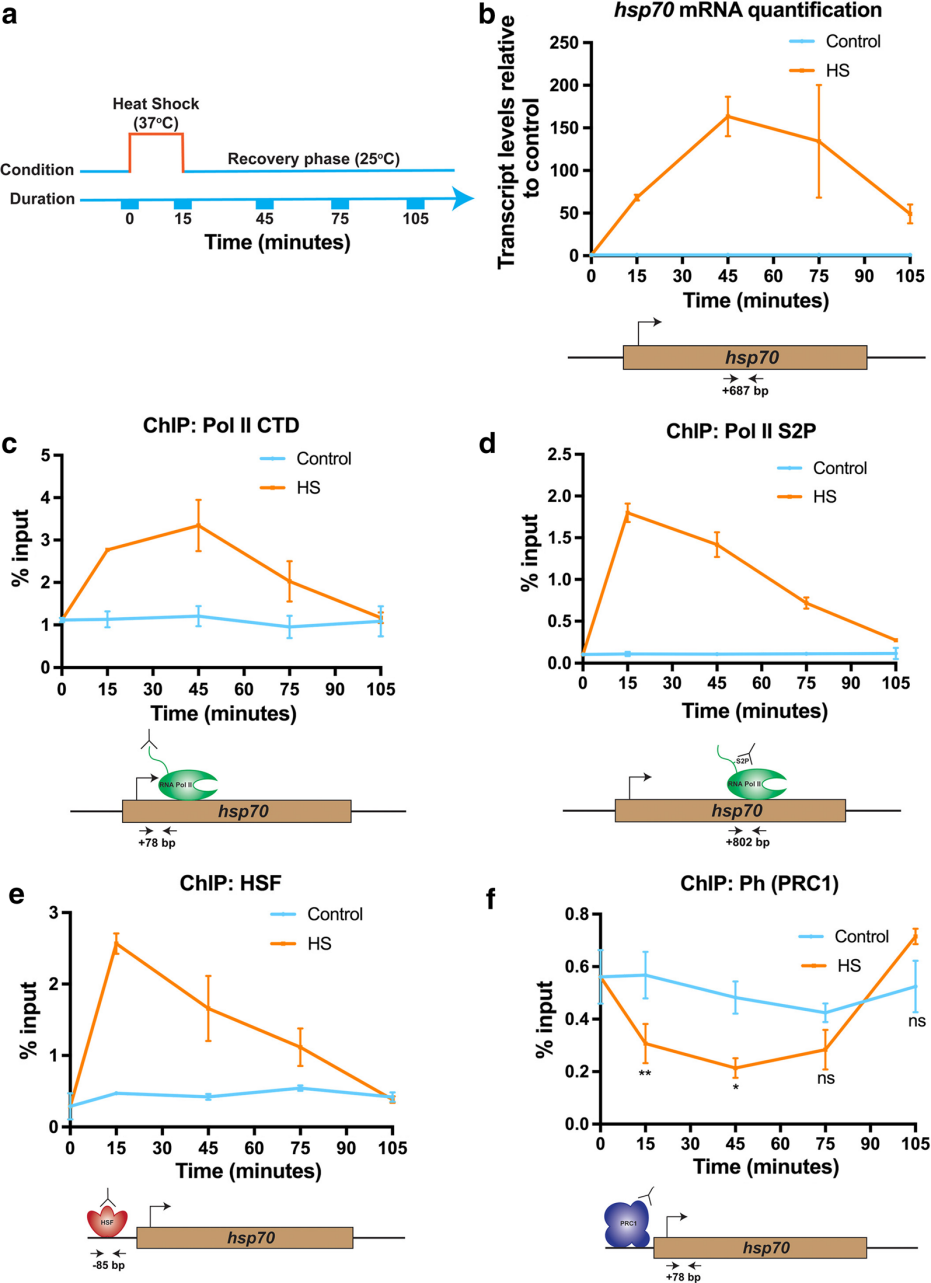
Antagonistic binding patterns of HSF and PRC1 at the *hsp70* locus. **a** Experimental scheme used throughout the study to measure the dynamics of mRNA/nascent RNA production, chromatin binding patterns of proteins and protein–protein interactions during the heat shock response. S2 DRSC cells were heat shocked at 37 °C for 15 min and thereafter allowed to recover from the heat shock at 25 °C for up to 90 min. At time points indicated in the scheme, cells were harvested and used for the following experimental procedures: mRNA quantification, ChIP-qPCR, co-IP and nascent RNA quantification. **b** Quantitative RT-PCR (qRT-PCR) detection of *hsp70* transcript levels during the heat shock response detailed in **a**. Distance of the location of the primers used from the *hsp70* transcription start site (TSS) is + 687 bp and has been depicted in the form of a cartoon below the data figure. The control line represents cells that were maintained at 25 °C for the entire duration of the time course detailed in **a**. **c**–**f** ChIP-qPCR measurements of occupancy levels of RNA polymerase II CTD, S2P form of RNA polymerase II, heat shock binding factor (HSF) and Polyhomeotic (Ph, a component of PRC1) at the *hsp70* locus during the heat shock response detailed in **a**. Distances of the location of the primers used from the *hsp70* TSS are as follows: for **c** and **f** (+ 78 bp), for **d** (+ 802 bp) and for **e** (− 85 bp) has been depicted in the form of a cartoon below the data figure. The control line represents cells that were maintained at 25 °C for the entire duration of the time course detailed in **a**. Data information: In **b**–**f**, data are presented as mean ± SEM for *n* = 2 (except for **f** where *n* = 3). Statistical significance was determined by performing a paired two-tailed *t* test, **P ≤ 0.01, *P ≤ 0.05 and ns = non-significant

To characterise the transcriptional and chromatin dynamics of the heat shock response, we focused on the binding kinetics of RNA polymerase II (Pol II), HSF and Polyhomeotic (Ph, a component of PRC1) at the *hsp70* locus using *Drosophila* S2 tissue culture cells. Our analysis revealed a rapid increase in *hsp70* transcripts immediately upon heat shock, followed by a steady decrease in mRNA levels as the locus is re-repressed, during the recovery phase in the absence of the heat shock stimulus (Fig. 1b). We investigated the binding dynamics of two forms of Pol II, the unphosphorylated C-terminal domain (CTD) form and the hyperphosphorylated (S2P) form of Pol II, at the promoter-proximal region and within the gene body of the *hsp70* locus, respectively. We detected a rapid increase in both forms of Pol II at the promoter-proximal region and within the gene body of the *hsp70* locus upon heat shock (Fig. 1c, d). This was accompanied by a decrease in Pol II binding levels at the *Act42A* gene (Additional file 2: Figure S2a, b), which is in accordance with the previously described phenomenon of the global decrease in Pol II levels genome-wide in *Drosophila* upon heat shock [22]. However, levels of bound Pol II at the *hsp70* locus steadily decreased as the cells recovered from the heat shock, with the locus returning to the paused state 90 min into the recovery phase (Fig. 1c, d). In contrast, Pol II binding at the *Act42A* gene increased over the recovery phase, indicating that the cells resumed a normal transcriptional programme within 90 min of the heat shock stimulus (Additional file 2: Figure S2a, b).

HSF binding is critical to release paused Pol II into productive elongation at the *hsp70* locus [23]. In agreement, we detected a rapid increase in HSF binding upon heat shock followed by steady decrease as the cells recovered from the heat shock (Fig. 1e). Thus, the chromatin binding dynamic of Pol II and HSF during the heat shock response was similar, emphasising a role for HSF binding at chromatin for the transcriptional activation of the *hsp70* locus [23].

Recent ChIP-seq studies in *Drosophila* and mammalian cell lines have revealed PRC1 binding at genes regulated by paused Pol II, leading to the hypothesis that PRC1 could regulate paused genes [11, 24, 25]. We, therefore, assessed the binding dynamics of Ph during the heat shock response. In keeping with PRC1’s role as a repressor, Ph occupancy at the *hsp70* locus decreased significantly upon activation. However, as the locus recovered from the heat shock and occupancy of Pol II and HSF decreased, Ph binding to chromatin was steadily restored to pre-activation levels (Fig. 1f).

Taken together, these results reveal an antagonism between HSF and Ph (PRC1) for occupancy at the *hsp70* locus.

### Co-localisation of Pleiohomeotic (Pho) and Pol II at the heat shock locus is independent of transcriptional elongation

**Fig. 2:**
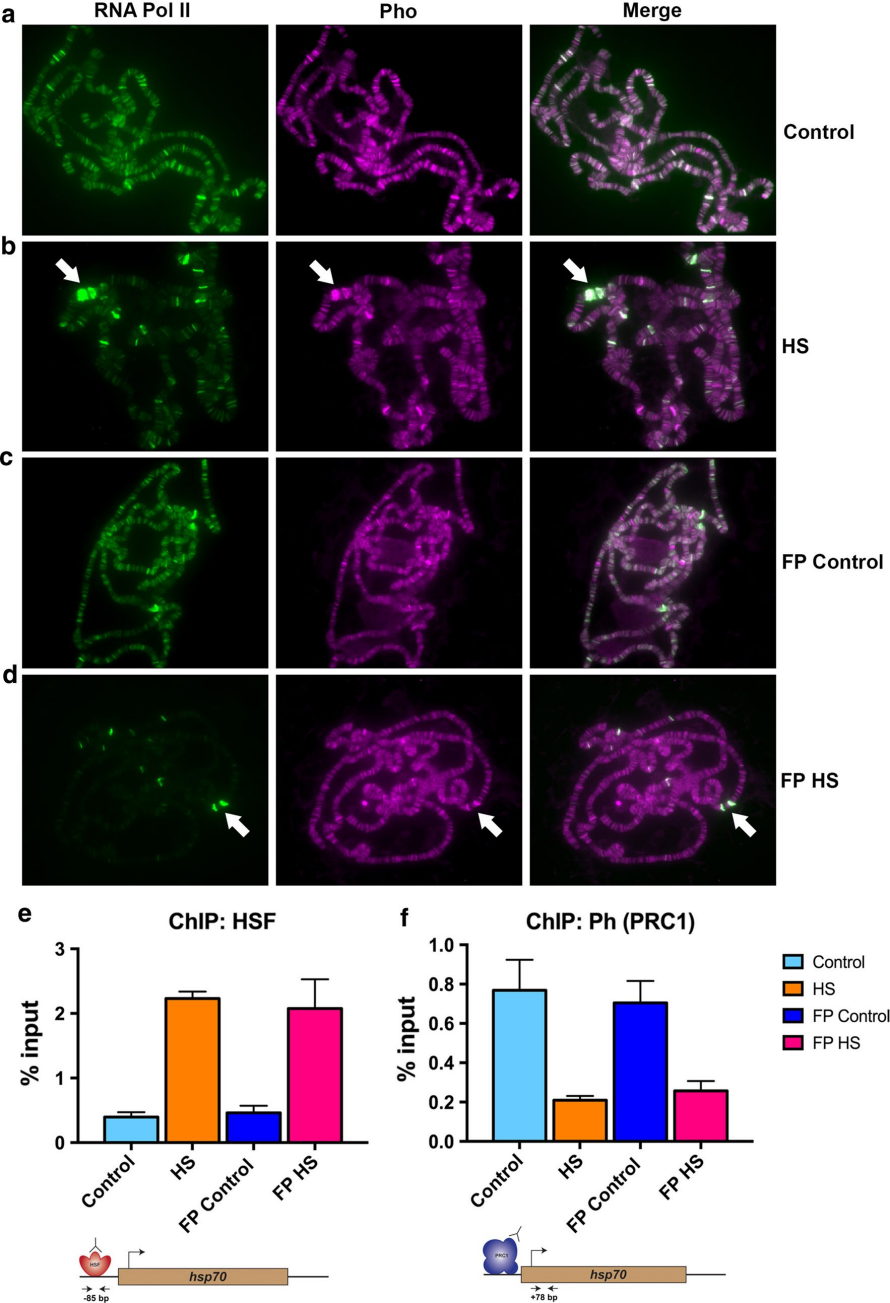
Co-localisation of Pleiohomeotic (Pho) and RNA polymerase II at the heat shock loci upon activation is independent of transcriptional elongation. **a**–**d** Double polytene immunostaining for RNA polymerase II (green) and Pho (magenta) under control, HS (heat shock), FP (flavopiridol) control and FP HS (flavopiridol + heat shock), respectively. The white block arrows point at the double heat shock puffs (87A/C) that were observed under HS and FP HS conditions. Magnification used 63×. **e**, **f** ChIP-qPCR measurements of occupancy levels for HSF and Ph, respectively, at the *hsp70* locus in S2 DRSC cells. Distances of the location of the primers used from the *hsp70* TSS are as follows: for **e** (− 85 bp) and for **f** (+ 78 bp) has been depicted in the form of a cartoon below the data figure. The control bar represents cells that were maintained at 25 °C for 15 min, the HS bar represents cells that were maintained at 37 °C for 15 min, the FP control bar represents cells that were treated with 500 nM flavopiridol for 40 min and then maintained at 25 °C for 15 min and the FP HS bar represents cells that were treated with 500 nM flavopiridol for 40 min and then maintained at 37 °C for 15 min. Data information: In **e**, **f**, data are presented as mean ± SEM for *n* = 2

We confirmed the previously identified co-localisation of Pho with Pol II at active genes by co-staining these factors on polytene chromosomes from larval salivary glands. Pho and Pol II co-localised at the majority of bands under basal conditions (Fig. 2a). Upon heat shock, Pol II binding was most prominent at the heat shock puffs (Fig. 2b). In addition, Pol II signal intensity decreased outside the heat shock loci, in accordance with a genome-wide decrease in Pol II occupancy upon heat shock (Fig. 2b). Interestingly, we observed a striking co-localisation of Pho and Pol II at all activated heat shock genes (Fig. 2b).

To determine whether this co-localisation is dependent upon transcription, we blocked transcriptional elongation by treatment with flavopiridol, a P-TEFb inhibitor, prior to heat shock [26]. The striking overlap in binding patterns of Pho and Pol II was not perturbed by flavopiridol treatment, suggesting that the interaction between these two factors could occur independently of transcriptional elongation (Fig. 2c). Furthermore, inhibition of P-TEFb followed by heat shock resulted in a marked decrease in the size of the heat shock puffs (Fig. 2d) and strongly reduced *hsp70* transcript levels (Additional file 3: Figure S3d). However, co-localisation of Pho and Pol II was not perturbed by flavopiridol (Fig. 2d). This result indicated that the co-localisation between Pho and Pol II was intact during the paused state as well as during the elongation phase of the transcriptional cycle. It is conceivable that Pho might co-localise with the paused form of Pol II. However, we found it intriguing that a DNA-binding protein like Pho was also capable of co-localising with actively elongating Pol II at chromatin. In order to understand whether the interaction between Pho and elongating Pol II was dependent upon the DNA-binding activity of Pho, we employed native ChIP (N-ChIP) qPCR to measure the occupancy levels of Pho over the time course detailed in Fig. 1a. We employed N-ChIP over formaldehyde cross-linked ChIP to measure the occupancy levels of Pho since N-ChIP only detects proteins that are associated with chromatin due to their direct interaction with DNA [27]. Indeed, while Pho was bound at the *hsp70* locus under control conditions, its occupancy decreased upon heat shock. However, as the cells recovered from the heat shock, the binding of Pho to the *hsp70* locus via DNA was restored (Additional file 3: Figure S3a). This suggested that the association of Pho to chromatin upon heat shock cannot be explained by its DNA-binding ability and could possibly be due to a protein–protein interaction with RNA Pol II or an elongation factor.

Previous observations established that flavopiridol treatment leads to considerable decrease in mature *hsp70* transcript production, even though the density of Pol II in the gene body remains normal [28]. We have now shown that chromatin binding levels of Ph, a component of PRC1, anti-correlate with mature *hsp70* transcript levels (Fig. 1f). Thus, we hypothesised that HSF recruitment in the presence of flavopiridol could be used to uncouple its effect on the occupancy of Ph at chromatin from the production of mature *hsp70* transcripts.

We, therefore, examined the consequence of flavopiridol treatment on the binding of PRC1 at the *hsp70* locus. We treated cells with flavopiridol prior to heat shock (hereafter referred to as FP + HS) followed by ChIP-qPCR. In agreement with previous observations, flavopiridol treatment led to a significant decrease in S2P Pol II levels in the *hsp70* gene body upon heat shock (Additional file 3: Figure S3c), whereas unphosphorylated Pol II and HSF binding levels were not perturbed (Additional file 3: Figure S3b and Fig. 2e). Ph binding at the *hsp70* locus decreased under FP + HS conditions, once again demonstrating that HSF and PRC1 binding patterns are mutually exclusive at the *hsp70* locus (Fig. 2e, f).

These data revealed that the co-localisation between Pho and Pol II was intact during the paused as well as during the elongation phase of the transcriptional cycle. Interestingly, while Pho continued to localise at the *hsp70* locus upon heat shock, Ph (PRC1) was evicted from the locus. We, thus, concluded that HSF binding could be capable of disrupting the ability of Pho to recruit PRC1 at the *hsp70* locus.

### Pho dynamically interacts with Spt5 and dSfmbt

**Fig. 3:**
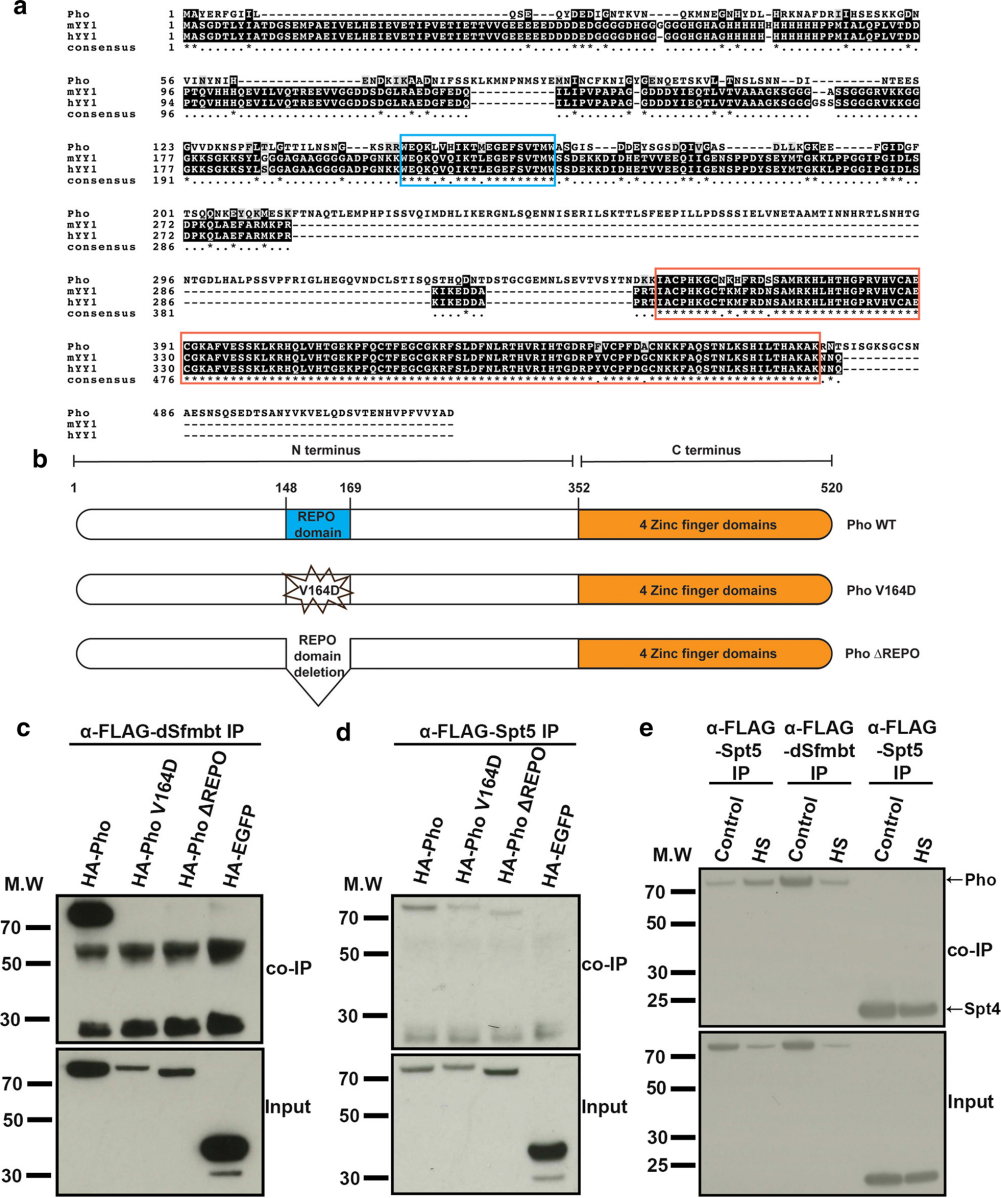
Pho interacts dynamically with Spt5 and dSfmbt. **a** Multiple protein alignment between Pho and YY1, the mouse and human homologue of Pho. The REPO domain is surrounded by a blue rectangle, while the Zn-finger domains are surrounded by an orange rectangle. The asterisk mark on the bottom line ‘consensus’ denotes the amino acid residues that are fully conserved between the three species. **b** Representation of the N- and C-termini of Pho and the mutant constructs generated for this study. **c**, **d** co-IP assays of S2 DRSC cells transiently transfected with plasmids expressing FLAG-tagged dSfmbt or Spt5 (FLAG-dSfmbt, FLAG-Spt5) and HA-tagged Pho, PhoV164D, Pho ΔREPO, EGFP (HA-Pho, HA-PhoV164D, HA-Pho REPO del, HA-EGFP) as indicated. Cell lysates were used for pull-downs using an anti-FLAG antibody and were later probed by Western blot using an anti-HA antibody. MW = molecular weight in kDa. **e** co-IP assays of S2 DRSC cells transiently transfected with plasmids expressing FLAG-tagged dSfmbt or Spt5 (FLAG-dSfmbt, FLAG-Spt5) and HA-tagged Pho or Spt4 (HA-Pho, HA-Spt4) as indicated. S2 DRSC cells were either maintained at normal growth temperature (25 °C) or heat shocked at 37 °C for 15 min, cell lysates were used for pull-downs using an anti-FLAG antibody and were later probed by Western blot using an anti-HA antibody. MW = molecular weight in kDa

Since we determined that the co-localisation of Pho and Pol II at the *hsp70* locus upon activation was independent of the DNA-binding ability of Pho, we hypothesised that this observation could possibly be explained by the reported genetic and biochemical interaction between Pho and Spt5 [20]. We also determined by ChIP-qPCR that chromatin occupancy of Spt5 does not change dramatically over the time course detailed in Fig. 1a (Additional file 4: Figure S4a). In order to gain mechanistic insight into the significance of the interaction between Pho and Spt5, we sought to identify the amino acid residues in Pho that mediate the protein–protein interaction with Spt5. Pho exhibits a modular structure with its protein–protein interaction domains localised in the N-terminus and its DNA-binding property ascribed to the Zn-finger domains in the C-terminus [5, 29, 30]. To identify putative Spt5-interacting regions of Pho, we searched for evolutionary conserved functional domain(s) by performing a multiple protein alignment between Pho and YY1, the mouse and human homologue of Pho. The Zn-finger domains in Pho and YY1 showed a high degree of conservation in the multiple protein alignment (Fig. 3a). However, a second stretch of amino acids, located in the N-terminus of Pho, also showed conservation with YY1 in the multiple protein alignment (Fig. 3a). This region mapped to a domain known as the Pho spacer region [5], and its homologous region within YY1 is known as the Recruitment of Polycomb (REPO) as it was shown to be necessary and sufficient for PcG-mediated repression in *Drosophila* [31].

We hypothesised that the REPO domain of Pho could be involved in mediating its protein–protein interaction with Spt5. To test this hypothesis, we generated two mutant constructs with the aim of disrupting the structure of the REPO domain [5] and analysed the effect of the mutation on the protein–protein interactions of Pho by co-immunoprecipitation (co-IP). The first construct, PhoV164D, contained a point mutation resulting in a valine (a hydrophobic amino acid residue) to aspartic acid (a hydrophilic amino acid residue) substitution to interfere with its ability to contact hydrophobic surfaces, while the second construct, ΔREPO, carried a 66-nucleotide deletion in the REPO domain [5] (Fig. 3b).

In agreement with previous findings, both mutant Pho constructs were incapable of interacting with dSfmbt [5] (Fig. 3c). However, both mutant Pho constructs also significantly impaired the interaction between Pho and Spt5, indicating that the REPO domain represents an interaction surface with multiple protein interaction partners (Fig. 3d). Next, we sought to explain the residual interaction between Pho and Spt5 in the mutant background using different N-terminal and C-terminal Pho constructs (1-351a.a N-Pho, 1-200a.a N-Pho, 173-351a.a N-Pho, 351-520a.a C-Pho). Our analysis revealed that in addition to the REPO domain containing N-terminal fragments of Pho, the C-terminus of Pho could also interact with Spt5, thus explaining the residual interaction in the absence of a functional REPO domain (Additional file 4: Figure S4c). In contrast, the Pho–dSfmbt interaction was solely dependent on the REPO domain (Additional file 4: Figure S4b). In conclusion, the REPO domain facilitated the interaction of Pho with dSfmbt (a PcG protein) as well as with Spt5 (a transcription elongation factor). These data led us to hypothesise that the protein–protein interactions of Pho might be dynamic under ON/OFF transcriptional states.

To test this hypothesis, we performed co-IPs of Pho–Spt5 and Pho–dSfmbt under control and heat shock conditions. Upon heat shock, we observed a considerable increase in the interaction between Pho and Spt5 and a concomitant reduction in the interaction between Pho and dSfmbt, which was stronger in non-stimulated cells (Fig. 3e and Additional file 4: Figure S4d). As a negative control, we tested whether the interaction between Spt4 and Spt5 is also dynamic upon heat shock. However, the interaction between Spt4 and Spt5 appeared stable under control and heat shock conditions (Fig. 3e). Thus, our co-IP experiments support the view that Pho can dynamically interact with Spt5 and dSfmbt depending upon the transcriptional state of the *hsp70* locus.

**Fig. 4:**
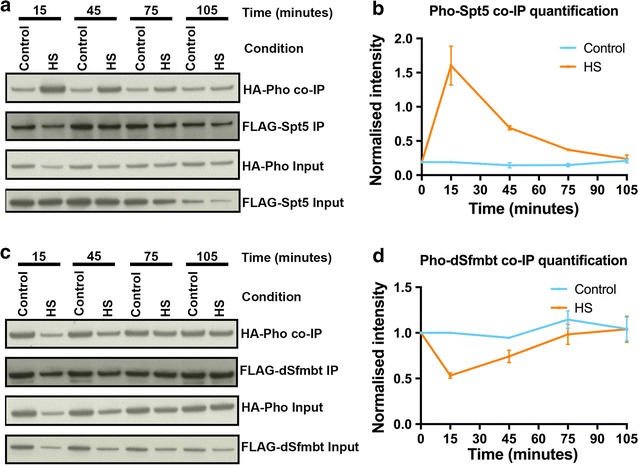
Pho preferentially interacts with Spt5 and dSfmbt during the active and silencing phase, respectively, of the heat shock response. **a**, **c** co-IP assays of S2 DRSC cells transiently transfected with plasmids expressing FLAG-tagged dSfmbt or Spt5 (FLAG-dSfmbt, FLAG-Spt5) and HA-tagged Pho (HA-Pho). The S2 DRSC cells were subjected to a heat shock and then allowed to recover from the heat shock according to the experimental scheme depicted in Fig. 1a. At the indicated time points in the experimental scheme, cell lysates were prepared and were used for pull-downs using an anti-FLAG antibody. They were later probed by Western blot using an anti-HA antibody. **b**, **d** Quantification of the co-IP assays in A and C. The control line represents cells that were maintained at 25 °C for the entire duration of the time course detailed in Fig. 1a. Details of the procedure used for quantification can be found in Materials and Methods. Data information: In **b** and **d**, data are presented as mean ± SEM for *n* = 2

Next, we tested whether the dynamic protein–protein interactions of Pho recapitulated the dynamic chromatin binding patterns of activators and repressors during the heat shock response. To this end, we analysed Pho–Spt5 and Pho–dSfmbt interactions during the heat shock response. Interestingly, the Pho–Spt5 interaction followed the Pol II and HSF (activator) binding dynamic. In contrast, the interaction between Pho and dSfmbt closely resembled the dynamic exhibited by Ph (PRC1, repressor) (Fig. 4a–d). This result suggested the possibility of a relationship between the chromatin binding dynamic of HSF and the dynamic protein–protein interaction of Pho with an activator (Spt5).

### HSF recruitment to chromatin is necessary and sufficient for the dynamic interaction between Pho and Spt5

Since our data suggested that HSF binding kinetics at the *hsp70* locus could influence the protein–protein interaction between Pho and Spt5, we asked whether HSF recruitment to chromatin would be sufficient and necessary for this dynamic protein–protein interaction. We, thus, relied on heat shock-independent ways to recruit HSF to chromatin.

**Fig. 5:**
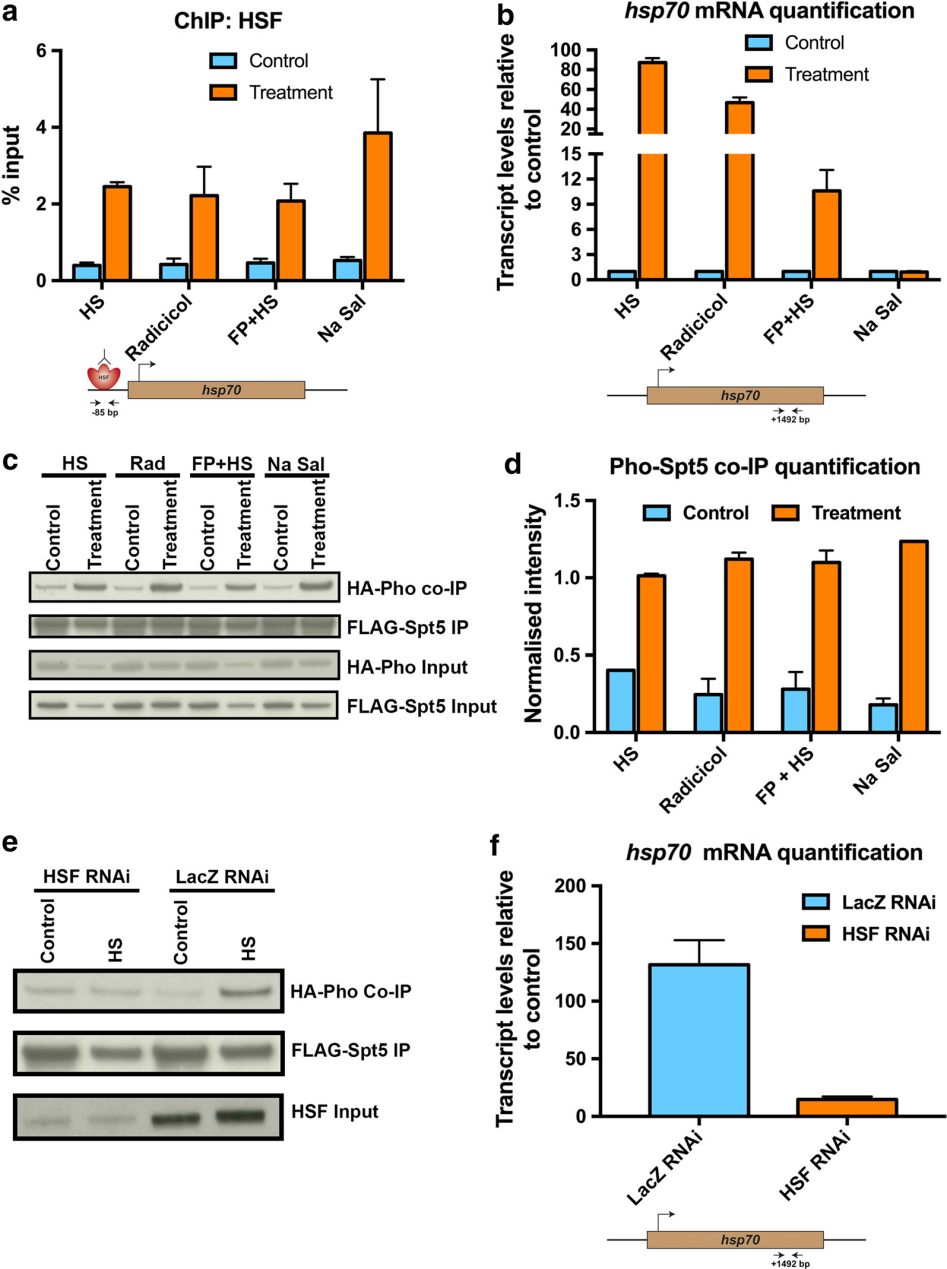
HSF recruitment to chromatin is necessary and sufficient for the dynamic protein–protein interaction between Pho and Spt5. **a** ChIP-qPCR measurements for the occupancy levels of HSF at the *hsp70* locus in S2 DRSC cells upon HS (heat shock), radicicol, FP + HS (flavopiridol + heat shock) and Na Sal (Sodium Salicylate) treatment conditions, respectively. In case of radicicol treatment, the control condition was DMSO treatment. For FP + HS, the control condition was FP control and for Na Sal, the control condition was nuclease-free water. Distance of the location of the primers used from the *hsp70* TSS is − 85 bp and has been depicted in the form of a cartoon below the data figure. **b** qRT-PCR measurements of *hsp70* transcript levels in S2 DRSC cells under HS, radicicol, FP + HS and Na Sal treatment conditions, respectively. Distance of the location of the primers used from the *hsp70* TSS is + 1492 bp and has been depicted in the form of a cartoon below the data figure. **c** co-IP assays of S2 DRSC cells transiently transfected with plasmids expressing FLAG-tagged Spt5 (FLAG-Spt5) and HA-tagged Pho (HA-Pho). The S2 DRSC cells were subjected to HS, radicicol, FP + HS and Na Sal, respectively; cell lysates were used for pull-downs with an anti-FLAG antibody and later probed by Western blot with an anti-HA antibody. **d** Quantification of the co-IP assays in **c**. Details of the procedure used for quantification can be found in Materials and Methods. **e** co-IP assays of S2 DRSC cells transiently transfected with plasmids expressing FLAG-tagged Spt5 (FLAG-Spt5) and HA-tagged Pho (HA-Pho). Cells were either treated with HSF or LacZ RNAi for 4 days, then either maintained at control conditions or heat shocked, cell lysates were prepared and used for pull-downs using an anti-FLAG antibody and later probed by Western blot using an anti-HA antibody. **f** qRT-PCR measurements of *hsp70* transcript levels in S2 DRSC cells under HSF and LacZ RNAi conditions. Distance of the location of the primers used from the *hsp70* TSS is + 1492 bp and has been depicted in the form of a cartoon below the data figure. Data information: In **a** and **b**, data are presented as mean ± SEM for *n* = 2, while in **e**, data are presented as mean ± SEM for *n* = 3

HSF is canonically chaperoned by Hsp90, which prevents it from trimerising and assuming its active conformation [32]. However, Hsp90 inhibition by radicicol triggers HSF to acquire its active confirmation through trimerisation, thus binding to its cognate site in the *hsp70* locus and causing transcriptional activation. We confirmed both recruitment of HSF to the *hsp70* locus by ChIP-qPCR and transcriptional activation of the locus upon radicicol treatment (Fig. 5a, b). Interestingly, the interaction between Pho and Spt5 was enhanced by Hsp90 inhibition (Fig. 5c, d). Therefore, we set out to decouple this increase in interaction from the transcriptional activation of the *hsp70* locus by recruiting HSF under conditions that caused little or no transcription from the *hsp70* locus.

Upon flavopiridol treatment followed by heat shock (FP + HS), HSF was recruited to the *hsp70* locus, but inhibition of P-TEFb led to a significant decrease in *hsp70* transcript levels (Fig. 5a, b). However, under FP + HS conditions, the interaction between Pho and Spt5 was still enhanced (Fig. 5c, d). Treatment with sodium salicylate has also been shown to recruit HSF to chromatin without affecting transcription from the *hsp70* locus [33]. While HSF was robustly recruited to chromatin upon sodium salicylate treatment, *hsp70* transcript levels remained unchanged when compared to the control condition (Fig. 5a, b). Upon sodium salicylate treatment, the interaction between Pho and Spt5 was, also, increased (Fig. 5c, d). We could, thus, uncouple the increase in interaction between Pho and Spt5 upon HSF recruitment from transcriptional activation. Next, we tested whether HSF binding was also necessary for this interaction switch between Pho and Spt5 by RNAi-mediated knockdown of HSF. By knocking down HSF, the dynamic nature of the Pho–Spt5 protein–protein interaction was undetectable when compared to the control LacZ RNAi condition (Fig. 5e, f).

Thus, by performing gain-of-function and loss-of-function experiments, we could establish that recruitment of HSF to chromatin is necessary and sufficient for the dynamic protein–protein interaction switch between Pho and Spt5.

In order to confirm that the protein–protein interaction between Pho and Spt5 was independent of the DNA-binding ability of Pho, we performed a co-IP between Pho and Spt5 under *DNase I* conditions. Indeed, we could confirm that the interaction as well as the dynamic switch between Pho and Spt5 was independent of DNA (Additional file 5: Figure S5a). We further went on to confirm the DNA-independent nature of the Pho–Spt5 interaction by performing a co-IP between N-Pho (lacking the DNA-binding Zn-finger domains) and Spt5 (Additional file 5: Figure S5b). However, we did observe dependence for the dynamic interaction switch between Pho and Spt5 on RNA binding (Additional file 5: Figure S5a). This observation is in line with our conclusion that HSF is necessary and sufficient for the dynamic interaction switch between Pho and Spt5 since HSF binding can trigger release of paused RNA Pol II leading to the production of nascent RNA transcripts [23]. Furthermore, the KOW4-5 domain of Spt5 has been shown to be capable of binding RNA and thereby influence the position of paused RNA Pol II [34].

### Absence of a functional REPO domain disrupts the transcriptional kinetics of the *hsp70* locus during the heat shock response

We next tested the effect of mutating the REPO domain on the dynamic protein–protein interaction between Pho and Spt5 during the heat shock response. To this end, we performed a co-IP for Pho V164D–Spt5 over the time course detailed in Fig. 1a and compared it to the Pho WT–Spt5 protein–protein interaction dynamic.

**Fig. 6:**
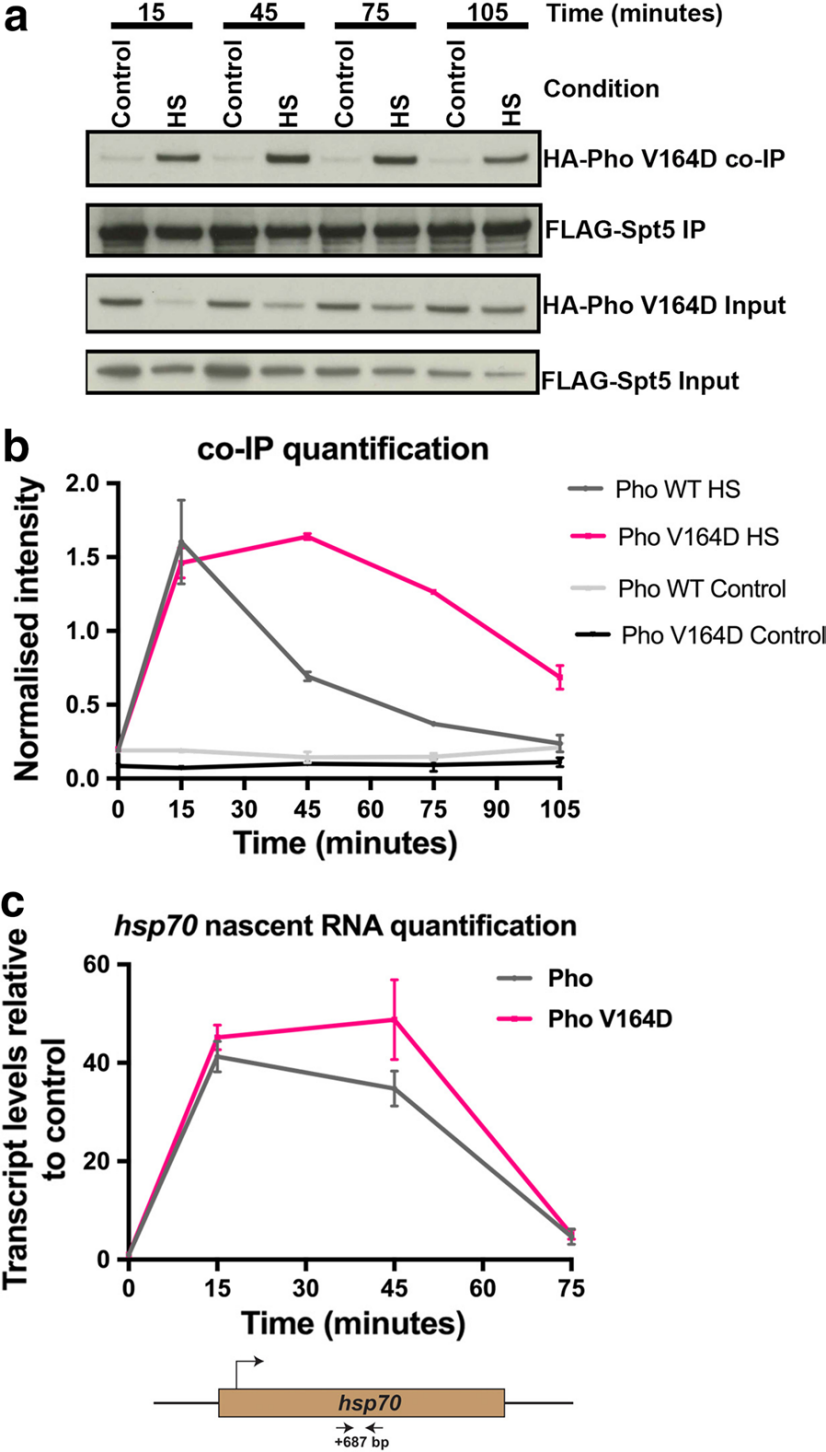
Absence of a functional REPO domain disrupts the transcriptional kinetics of the *hsp70* locus during the heat shock response. **a** co-IP assays of S2 DRSC cells transiently transfected with plasmids expressing FLAG-tagged Spt5 (FLAG-Spt5) and HA-tagged PhoV164D (HA-PhoV164D). The S2 DRSC cells were subjected to a heat shock and then allowed to recover from the heat shock according to the experimental scheme depicted in Fig. 1a. At the indicated time points in the experimental scheme, cell lysates were prepared and were used for pull-downs using an anti-FLAG antibody. They were later probed by Western blot using an anti-HA antibody. **b** Quantification of the co-IP assay in **a** and its comparison with the quantification of the Pho–Spt5 co-IP assay across the same time course. The control lines represent cells that were maintained at 25 °C for the entire duration of the time course detailed in Fig. 1a. Details of the procedure used for quantification can be found in Materials and Methods. **c** qRT-PCR measurements of nascent *hsp70* transcripts produced over the course of the heat shock response from S2 DRSC cells upon over-expression of WT Pho and mutant Pho (PhoV164D), respectively. Distance of location of the primers from the *hsp70* TSS is + 687 bp and has been depicted in the form of a cartoon below the data figure. Data information: In **b**, data are presented as mean ± SEM for *n* = 2, while in **c**, data are presented as mean ± SEM for *n* = 3

To our surprise, mutant and wild-type Pho exhibited comparable interaction strengths with Spt5 upon heat shock, indicating that the dynamic interaction switch upon heat shock is not affected by the V164D point mutation in the REPO domain (Fig. 6a, b). However, while the interaction between Pho WT–Spt5 diminished as the *hsp70* locus was repressed in the absence of the activating stimulus, the increase in interaction between Pho V164D–Spt5 remained sustained during the recovery phase (Fig. 6a, b). This could be explained by the fact that in the absence of a functional REPO domain, dSfmbt could no longer compete with Spt5 for interaction with Pho during the recovery phase of the heat shock response (Fig. 6a, b). Similar to the Pho WT–Spt5 interaction dynamic, the Pho V164D–Spt5 interaction switch was independent of DNA but dependent upon RNA (Additional file 6: Figure S6a).

To directly assess the effect of the PhoV164D mutant on transcription from the *hsp70* locus upon heat shock, we expressed the PhoV164D mutant by placing it under the control of the copper-inducible metallothionein promoter. As a control, PhoWT was expressed under the control of the same promoter. The expression levels of the copper-inducible constructs were compared to endogenous Pho after 72 h of induction (Additional file 6: Figure S6b). After expression of the WT and mutant constructs for 72 h, respectively, the transcriptional kinetics of the heat shock response was assessed by measuring nascent RNA production from the *hsp70* locus. The expression of the PhoV164D mutant did not affect the up-regulation of the *hsp70* locus in response to a heat shock as compared to the control (Fig. 6c). However, upon withdrawing the heat shock stimulus, while nascent RNA production in the control cells sharply decreased, this was not observed in the case of PhoV164D mutant (Fig. 6c). Thus, the absence of a functional REPO domain negatively affected the recovery kinetics of the *hsp70* locus during the heat shock response.

## Discussion

Our study aimed at gaining mechanistic understanding about how PcG proteins regulate their target genes, which are transiently activated by an extracellular stimulus. To this end, we used the heat shock-responsive *hsp70* gene in *Drosophila* as it exhibits a dynamic pattern of expression during the well-characterised heat shock response and it is also a target for PRC1. Furthermore, mechanistic analysis supports a role for PcG proteins in the re-establishment of silencing at the *hsp70* locus in the absence of the heat shock stimulus.

Our results highlight antagonism between HSF and PRC1 for binding at the *hsp70* locus, suggesting a role for PRC1 in repressing the locus in the absence of the heat shock stimulus. Furthermore, we could uncouple the eviction of PRC1 from the *hsp70* locus upon heat shock from production of mRNA by treating cells with flavopiridol, a P-TEFb inhibitor. We demonstrated that Pho could preferentially interact with Spt5 and dSfmbt under ON/OFF conditions, respectively, at the *hsp70* locus. Our data also conclusively showed that the recruitment of HSF to chromatin is sufficient and necessary for the dynamic interaction between Pho and Spt5. The REPO domain of Pho is central for the interaction switch, since a mutation in the REPO domain not only disrupted the kinetics of the protein–protein interaction between Pho and Spt5 but also disrupted the kinetics of nascent RNA production from the *hsp70* locus during the heat shock response.

Several reports have described a role for Pho in both the activation and the repression of its target genes [19, 35, 36]. In order to dissect the relationship between binding patterns of PRC1, Pho and Trx and their respective effect on transcriptional activity, ChIP-chip was employed to identify their binding sites in the ANT-C and BX-C gene clusters in two different *Drosophila* cell lines. At silent loci, the Pho signal localised at PREs. However, in case of active loci, the Pho signal spread over the gene body [19]. Another study demonstrated the requirement of a Pho-binding site and a functional Pho protein for a pairing-sensitive silencing element in the *Drosophila even skipped* (*eve*) locus for propagation of the silenced as well as the activated state in different cellular contexts, respectively [35]. Yet another reported example is the Ubx gene in *Ubx* ^ON^ and *Ubx* ^OFF^ cells in the developing *Drosophila* larvae; the levels of Pho remained unchanged at the *bx* PRE in *Ubx* ^ON^ cells, while the occupancy of PRC1 and PRC2 was reduced by twofold [36].

Our results demonstrate the ability of Pho to switch its protein interaction partners in a HSF-dependent manner. Our current observations at the *hsp70* locus lead us to propose that, in the absence of transcription factor binding, Pho interacts with dSfmbt, forming a recruitment platform for PRC1 and PRC2, and stably maintains the silent state [6, 9, 37]. However, upon activation, TF binding could lead to an increase in interaction affinity between Pho and Spt5, thereby relieving Polycomb-mediated silencing and enabling a switch to the activated state. It is thus plausible that dynamic protein–protein interaction switches between Polycomb group members and Spt5 could potentially play a role in enabling rapid switches in expression states at Pho target genes.

Our study also assessed the contribution of the dynamic switch in Pho protein–protein interactions on the transcriptional output of the *hsp70* locus during the heat shock response. It was notable that Pho preferentially interacts with Spt5 and dSfmbt when the *hsp70* locus is active and silent, respectively. A point mutation in the REPO domain of Pho resulted in ablation of its interaction with dSfmbt. While the PhoV164D mutant was capable of strongly interacting with Spt5, immediately upon heat shock, it was incapable of switching back to its interaction with dSfmbt in the absence of the heat shock stimulus. This result underscores the importance of a functional REPO domain in mediating the switch in the protein interaction partners of Pho depending upon the binding kinetics of HSF at the *hsp70* locus. Nascent RNA analysis also suggested that the dynamic protein–protein interactions of Pho could contribute to the transcriptional regulation of the *hsp70* locus. Expression of PhoV164D mutant negatively affected the transcriptional kinetics of the *hsp70* locus in response to a heat shock when compared to the control. We speculate that in the absence of a functional REPO domain, the dynamic nature of the protein–protein interactions of Pho is lost, thereby preventing cells from efficiently switching between ON/OFF states in the presence/absence of the activating stimulus.

Taken together, our study highlights how dynamic protein interaction switches between PcG members and Spt5 could facilitate the transcriptional regulation of a Polycomb-regulated inducible gene like *hsp70*. Additional open questions remain regarding the complex relationship between PcG proteins and paused RNA polymerase II. Nonetheless, this study provides a new perspective on how the plasticity of Polycomb gene silencing is mediated by dynamic protein–protein interaction switches between activators and repressors.

## Conclusions

In this study, we sought to understand the mechanistic basis of dynamic gene regulation by Polycomb group proteins at stress-inducible loci like *hsp70*. We identified a role for the dynamic protein–protein interaction switch between Pho and Spt5 in the transcriptional regulation of the *hsp70* locus. Crucially, we show that the recruitment of HSF to chromatin and a functional REPO domain in Pho is necessary for the dynamic interaction switch between Pho and Spt5. Thus, our study reveals how a dynamic interaction switch between PcG proteins and an elongation factor could enable inducible genes regulated by Polycomb to switch between active and repressed transcriptional states.

## Materials and methods

### Cell culture

S2 DRSC cells (DGRC, stock #181) were cultured in Schneider’s medium (Sigma-Aldrich) + 10% heat-inactivated FBS (PAN Biotech GmbH) at 25 °C.

Cells were instantaneously heat shocked by adding Schneider’s medium, which was pre-warmed to 48 °C, and then, the temperature was maintained at 37 °C for 15 min by incubating the cells in a water bath as previously described [18]. The cells were then placed into a water bath at 25 °C to enable recovery from the heat shock. In case of treatment with 40 μM radicicol, 500 nm flavopiridol and 10 μM sodium salicylate, the treatment was performed for 1 h, 40 and 10 min, respectively, at 25 °C.

### Chemicals and antibodies

The following chemicals were used in this study: flavopiridol (F3055, Sigma-Aldrich), radicicol (R-370, Alomone), sodium salicylate (S3007, Sigma-Aldrich), copper sulphate (102790, Merck).

The following primary antibodies were used in this study: α-mouse RNA polymerase II CTD repeat (Abcam, ab817), α-mouse RNA polymerase II CTD repeat phosphor S5 (Abcam, ab5408), α-rabbit RNA polymerase II CTD repeat phospho S2 (Abcam, ab5095), α-rat RNA polymerase II CTD repeat phosphor S2 (Active Motif, Cat#61083), α-rabbit HSF (gift from John Lis), α-rabbit Polyhomeotic (Paro lab stock), α-rabbit Pleiohomeotic (gift from Judith Kassis), α-rabbit Pleiohomeotic (gift from Jurg Muller), α-mouse FLAG (Sigma, F1804), α-rat HA (Roche, 11 867 423 001).

The following secondary antibodies were used in this study: α-mouse IgG HRP-linked whole antibody (GE Healthcare, NA931), α-rabbit IgG HRP-linked whole antibody (GE Healthcare, NA934V), α-rat IgG HRP-linked whole antibody (GE Healthcare, NA935V), Goat α-mouse IgG Alexa Fluor 488 (Thermo Fisher Scientific, A-11001), Goat α-rabbit IgG Alexa Fluor 568 (Thermo Fisher Scientific, A-11011).

### Gene cloning

cDNA clones for Spt5 (LD10265) and dSfmbt (LD14884) were obtained from the *Drosophila* Genome Resource Centre (DGRC). cDNA for Pho and Spt4 was prepared from S2 DRSC cells using the First Strand cDNA synthesis kit (Thermo Fisher Scientific, K1612).

In order to express the constructs in S2 DRSC cells, the cDNA clones were amplified using appropriate primers and cloned in pENTR/D-TOPO vector using the pENTR™/D-TOPO™ cloning kit (Thermo Fisher Scientific, K240020). The entry clones were subsequently cloned into pAFW, pAHW, pAWH, pMT/FHW (*Drosophila* Gateway Vector Collection by DGRC) by using the Gateway™ LR clonase™ II enzyme mix (Thermo Fisher Scientific, 11791020). All plasmids were sequenced by Sanger Sequencing (Microsynth AG). All primer sequences used in this study are listed in Additional file 7: Table S1.

### Cell Transfection

In case of transient co-transfection for co-IP experiments, 1 μg of each plasmid was used for transfection, and procedure was performed according to the Effectene transfection reagent protocol (Qiagen, 301425).

In case of stable transfection for generation of cell lines, 2 μg of pMT/FHW HygRes [38] was transfected into cells according to the Effectene transfection reagent protocol and cells were selected with Hygromycin B (Thermo Fisher Scientific, 10687010) 3 days post-transfection until the emergence of drug-resistant colonies was observed.

### RNAi

Sequences used for the production of double-stranded RNA against HSF and LacZ have been previously described [23]. The procedure of dsRNA production was performed according to the manufacturer’s protocol (MEGAscript™ RNAi kit, Thermo Fisher Scientific, AM1626).

Cells were treated with dsRNA according to the protocol by DRSC (Cell RNAi, 6-well) with the following exceptions. One million cells were plated in 1 ml of serum-free medium onto 6-well plates. Ten micrograms of dsRNA was added to the well, and this was followed by a 45-min incubation period. Thereafter, 1 ml of Schneider’s medium + 20% heat-inactivated FBS was added to the wells, and the cells were incubated for a period of 96 h.

### Chromatin immunoprecipitation

Approximately 25 × 10^6^ cells were resuspended in PBS and fixed with 1% formaldehyde (Sigma, F8775) for 10 min. The reaction was quenched with 125 mM glycine for 5 min. Cells were washed with ice-cold PBS and centrifuged at 500*g* for 5 min. The cells were resuspended in buffer A (100 mM Tris ph8, 10 mM DTT) and incubated at 4 °C on a rotor for 15 min. Thereafter, the cells were incubated on a thermomixer at 30 °C at 300 rpm. The cells were briefly vortexed for 5 s and centrifuged at 500*g* for 5 min. The cells were resuspended in buffer B (10 mM HEPES ph8, 10 mM EDTA, 10 mM EGTA, 0.25% Triton X-100) and incubated at 4 °C on a rotor for 5 min. The cells were centrifuged at 500*g* for 5 min. The cells were resuspended in buffer C (10 mM HEPES ph8, 10 mM EDTA, 0.5 mM EGTA, 200 mM NaCl) and incubated at 4 °C on a rotor for 5 min. The cells were centrifuged at 500*g* for 5 min. The cells were resuspended in buffer D (50 mM HEPES ph8, 10 mM EDTA, 0.5 mM EGTA, 0.1% SDS) and sonicated using S220 ultrasonicator (Covaris) to obtain chromatin fragments approximately 500 bp long.

The chromatin was adjusted to RIPA conditions and centrifuged at 4 °C for 10 min at 13,000 rpm. One hundred microlitres of the chromatin was aliquoted as an input fraction. Appropriate amount of the antibody (amount of each antibody used per IP is stated in Additional file 8: Table S2) was added to the chromatin solution and incubated overnight on a rotor at 4 °C.

In order to prepare the Dynabeads™ Protein A/G (Thermo Fisher Scientific) for purification of the immunocomplexes, 40 μl of the magnetic beads was washed with 1 ml RIPA. The ChIP sample was centrifuged at 4 °C for 5 min at 13,000 rpm, transferred to the tube containing the magnetic beads and incubated at 4 °C on a rotor for 2–3 h. The immunocomplexes along with the magnetic beads were washed five times with RIPA buffer (10 mM Tris ph8, 1 mM EDTA, 1% Triton X-100, 0.1% SDS, 0.1% Na-deoxycholate), once with LiCL buffer (10 mM Tris ph8, 250 mM LiCl, 1 mM EDTA, 0.5% NP40, 0.5% Na-deoxycholate) and twice with TE (10 mM Tris ph8, 1 mM EDTA). The immunocomplexes were eluted thrice with the elution buffer (1% SDS, 0.1 M NaHCO_3_) at 65 °C for 15 min with gentle agitation (800 rpm). The ChIP sample was reverse cross-linked with 200 mM NaCl and incubated at 65 °C overnight. The input and ChIP samples were treated with RNase (Roche, 11 119 915 001) and incubated at 37 °C for 90 min. This was followed by treatment with Proteinase K (Axon Lab AG, A4392.0001) and incubated at 55 °C for 90 min. The DNA was purified using QIAquick PCR purification kit (QIAGEN, 28106). All buffers were supplemented with protease inhibitors (cOmplete EDTA-free, Roche, 4693132001).

### Polytene immunostaining

*Drosophila* salivary glands were dissected from third-instar larvae, permeabilised in solution 1 (1xPBS, 1% Triton X-100) and then fixed for 10 min (50% acetic acid, 3.7% p-formaldehyde). The squashed salivary glands were prepared as described previously [39]. The slides were washed with PBS and blocked for 60 min with the blocking solution (3% BSA, 0.2% NP40, 0.2% Tween 20, 10% non-fat dry milk powder). Dilutions of the primary antibody (Pho, 1:50 and RNA pol II, 1:100) were made with the blocking solution, and the slides were incubated with the primary antibody overnight in a humid chamber at 4 °C. The slides were washed with PBS, dilutions of the secondary antibody (1:200) were made with the blocking solution and the slides were incubated with the secondary antibodies for 60 min in a humid chamber at room temperature. The slides were washed with wash buffer ‘300’ and wash buffer ‘400’ (1xPBS, 300/400 mM NaCl, 0.2% NP40, 0.2% Tween 20). The slides were then stained with 1 μg/ml DAPI (Roche, 236 276), mounted using 40 μl Fluoromount G (Southern Biotech, 0100-01) and analysed using a Leica DMI6000 B fluorescent microscope.

### Native ChIP (N-ChIP)

The protocol for N-ChIP had been previously described in [27]. Approximately 2 × 10^8^ cells were harvested by scraping, washed with ice-cold PBS and centrifuged at 1200*g* at 4 °C for 4 min. The cells were resuspended in TM2+ buffer (10 mM Tris pH 7.5, 2 mM MgCl_2_, 0.5 mM PMSF). Nuclei were released by adding 0.6% NP-40 to the cell suspension and incubating for 3 min and 30 s on ice. During the incubation, the cell suspension was gently vortexed at intervals of 1 min. The nuclei were centrifuged at 4 °C for 10 min at 1000*g* and washed once with TM2+. The nuclei were resuspended in TM2+ with protease inhibitors (cOmplete EDTA-free, Roche, 4693132001). MNase treatment was performed as follows: the nuclei were preheated at 37 °C for 3 min. 1 mM CaCl_2_ was added to the nuclei followed by addition of 80U MNase (M0247S, NEB). The digestion with MNase was carried out for precisely 6 min, and the reaction was stopped by addition of 2 mM EGTA. The nuclei were incubated on ice for 2 min after the MNase treatment and centrifuged at 4 °C for 10 min at 1000*g*. Nuclei were washed with TM2+ containing protease inhibitors and 2 mM EGTA and centrifuged at 4 °C for 10 min at 1000*g*. The nuclei were resuspended in 80T buffer (70 mM NaCl, 10 mM Tris pH 7.5, 2 mM MgCl_2_, 2 mM EGTA, 0.1% Triton X-100, 0.5 mM PMSF, protease inhibitors), cavitated 10 times through a 26G × ½” needle (4710004512, Henke Sass Wolf), and chromatin was centrifuged at 4 °C for 10 min at 1000*g*. Ten percentage of the chromatin was aliquoted as the input fraction. Anti-Pho antibody (rabbit polyclonal, a gift from Jurg Muller) was used for each IP at 1:100 dilution and incubated at 4 °C on a rotating wheel overnight. Forty microlitres of Dynabeads™ Protein G magnetic beads was added to the IP and incubated at 4 °C for 2 h. Beads were washed twice with 80T buffer, and beads were resuspended in 80T buffer. Following treatment with *RNase A* (Thermo Fisher Scientific, EN0531) and Proteinase K (Axon Lab AG, A4392.0001), DNA was extracted by phenol–chloroform and ethanol precipitation.

### Co-immunoprecipitation (co-IP)

The co-IP protocol was adapted from [40]. Cells were harvested, washed with ice-cold PBS and centrifuged at 4 °C for 5 min at 500*g*. The cells were resuspended and lysed in co-IP buffer (50 mM Tris ph7.5, 150 mM NaCl, 1% NP40, 1 mM EGTA, 20 mM NaF) for 30 min at 4 °C. The cells were centrifuged at 4 °C for 20 min at 12,000 rpm. Prior to treating the cell lysate with nucleases, 0.4 mM MgCl_2_ was supplemented to the cell lysate. The cell lysate was incubated with *RNase A* (Thermo Fisher Scientific, EN0531) or *DNase I* (Thermo Fisher Scientific, AM2222) for 10 min at room temperature. The reaction with the nuclease was terminated by addition of 5 mM EDTA to the cell lysate. The supernatant was transferred to a siliconised tube. Ten percentage was aliquoted as the input fraction. 2.5 μg of α-mouse FLAG was added to the IP fraction and incubated at 4 °C for 2–3 h. Forty microlitres of Dynabeads™ Protein G magnetic beads was added to the IP fraction and incubated at 4 °C for 2–3 h. The immunocomplexes were washed thrice with co-IP wash buffer (10 mM Tris ph7.5, 1 mM EDTA, 1 mM EGTA, 150 mM NaCl, 1% Triton X-100). The immunocomplexes were eluted with co-IP buffer, denatured with a mixture containing NuPage^®^ 4x LDS sample buffer (Thermo Fisher Scientific, NP0008) and 200 mM DTT upon heating to 70 °C with mild agitation at 800 rpm for 15 min. Western blots were visualised using Amersham ECL Western Blotting Detection reagent (GE Healthcare, RPN2106) and Amersham Hyperfilm ECL (GE Healthcare, 28906836). All buffers were supplemented with protease inhibitors (cOmplete EDTA-free, Roche, 4693132001) and phosphatase inhibitors (PhosSTOP phosphatase inhibitor cocktail tablets, Roche, 4906837001). For the quantification of HA-Pho co-IP, the intensity of the co-IP band was normalised against the intensity of the corresponding FLAG IP band using ImageJ software.

### Native elongating transcript isolation and quantification

The protocol used for the isolation of native elongating transcripts had been previously described [41]. Cells were grown in medium containing 0.5 mM CuSO_4_ for 72 h to induce the expression of stably integrated constructs. Cells were harvested and centrifuged at 2000 rpm for 5 min at 4 °C. The cells were resuspended in PBS and centrifuged at 2000 rpm for 5 min at 4 °C. The cells were resuspended in cytoplasmic buffer (10 mM Tris pH 7, 150 mM NaCl, 0.15% NP-40) and incubated on ice for 5 min. The cell lysate was layered on top of NET-seq sucrose buffer (10 mM Tris pH 7, 150 mM NaCl, 0.34 M sucrose) and centrifuged at 16,000*g* for 10 min at 4 °C. Nuclei were washed twice with nuclei wash buffer (1 mM EDTA, 0.05% Triton X-100, PBS) and centrifuged at 2000*g* for 5 min at 4 °C. The nuclei were resuspended in glycerol buffer (20 mM Tris pH 8, 75 mM NaCl, 0.5 mM EDTA, 50% glycerol, 0.85 mM DTT) and harsh nuclei lysis buffer (20 mM HEPES ph 8, 300 mM NaCl, 1% NP-40, 1 M urea, 0.2 mM EDTA, 1 mM DTT). This was followed by 4 rounds of vortexing for 2 s each with an incubation period of 1 min on ice between each round of vortexing. Samples were incubated on ice for 2 min and centrifuged at 18,500*g* for 2 min at 4 °C. The supernatant represented the nucleoplasmic fraction while the pellet contained the chromatin fraction. The chromatin fraction was washed once using a mix of the glycerol and harsh nuclei lysis buffer. The pellet was resuspended in 500 μl of TRIzol. All the buffers were supplemented with protease inhibitors (cOmplete EDTA-free, Roche, 4693132001) and actinomycin D (Sigma, A1410).

Nascent RNA was isolated from the chromatin fraction following manufacturer’s protocol (Thermo Fisher Scientific, 15596018). The RNA was precipitated overnight using ethanol. Mature RNAs were depleted from the sample by performing two rounds of Oligo dT-based depletion (Dynabeads™ Oligo(dT)_25_, Thermo Fischer Scientific, 61002). The RNA was DNase-treated using Turbo DNA-free™ kit (Thermo Fisher Scientific, AM1907) and reverse transcribed using First Strand cDNA synthesis kit (Thermo Fisher Scientific, K1612). cDNA was diluted (1:50), mixed with FastStart Essential DNA Green Master (Roche, 06402712001) and assayed using Light Cycler^®^ 96 (Roche). ΔΔC^T^ method was used to measure transcript levels relative to control.

### Quantitative real-time PCR

For measurement of mRNA levels using qRT-PCR, cells were lysed using TRIzol™ reagent (Thermo Fisher Scientific, 15596018), and RNA was extracted using Direct-zol™ RNA MiniPrep (Zymo Research, R2052). The RNA was DNase-treated using Turbo DNA-free™ kit (Thermo Fisher Scientific, AM1907) and reverse transcribed using First Strand cDNA synthesis kit (Thermo Fisher Scientific, K1612). cDNA was diluted (1:50), mixed with FastStart Essential DNA Green Master (Roche, 06402712001) and assayed using Light Cycler^®^ 96 (Roche). ΔΔC^T^ method was used to measure transcript levels relative to control.

For measurement of % input values for the ChIP and N-ChIP samples, the input DNA samples were diluted in fivefold dilution series (1:10 up to 1:1250) and the ChIP sample was diluted (1:5). The standard curve obtained from the input DNA dilutions was used to determine the amount of DNA in the ChIP, which was calculated in terms of % input.

## Additional files

**Additional file 1: Figure S1.** Schematic representation of the proteins involved in the silencing, activation and re-silencing of the *hsp70* locus. a At optimal growth temperature, Pho, a DNA-binding PcG member, binds to promoter region of the *hsp70* locus. Pho interacts with dSfmbt, which together form a recruitment platform for PRC1 to the *hsp70* locus. In addition, RNA polymerase II is maintained in the paused state by NELF and Spt5, which act as pausing factors. Upon heat shock, HSF, along with P-TEFb, is recruited to chromatin and releases RNA polymerase II from the paused state. P-TEFb modifies Spt5 and converts into an elongation factor. It also modifies the CTD of RNA polymerase II to enable productive elongation. However, upon removal of the heat shock stimulus, the locus should eventually return to its paused state. Thus, *hsp70* is an ideal model gene to study the eviction and recruitment of PRC1 upon activation and re-silencing, respectively. The colour code for the protein names is as follows: silencing in red, pausing factors in orange and activators in green.

**Additional file 2: Figure S2.** RNA polymerase II binding dynamics at *Act42A* locus during the heat shock response. a, b ChIP-qPCR measurements of occupancy levels of RNA polymerase II CTD and S2P form of RNA polymerase II at the *Act42A* locus during the heat shock response detailed in Fig. 1a. The control line represents cells that were maintained at 25 °C for the entire duration of the time course detailed in Fig. 1a. Distance of the location of the primers used from the *Act42A* TSS is as follows: for a and b (+ 104 bp) and has been depicted in the form of a cartoon below the data figure. Data information: In (a, b), data are presented as mean ± SEM for *n* = 2.

**Additional file 3: Figure S3.** Effect of flavopiridol on RNA polymerase II occupancy at the *hsp70* locus upon heat shock. a N-ChIP-qPCR measurement of the occupancy level of Pho at the *hsp70* locus in S2 DRSC cells during the heat shock response detailed in Fig. 1a. Distance of the location of the primers used from the *hsp70* TSS is − 85 bp and has been depicted in the form of a cartoon below the data figure. b, c ChIP-qPCR measurements of occupancy levels of RNA polymerase II CTD and S2P form of RNA polymerase II, respectively, at the *hsp70* locus in S2 DRSC cells. Distances of the location of the primers used from the *hsp70* TSS are as follows: for a (+ 78 bp) and for b (+ 802 bp) and has been depicted in the form of a cartoon below the data figure. d qRT-PCR measurements of *hsp70* transcript levels in third-instar *Drosophila* larvae under the conditions used for double polytene immunostaining. Distance of the location of the primers used from the *hsp70* TSS is + 687 bp and has been depicted in the form of a cartoon below the data figure. For b–d, the control bar represents cells that were maintained at 25 °C for 15 min, the HS bar represents cells that were maintained at 37 °C for 15 min, the FP control bar represents cells that were treated with 500 nM flavopiridol for 40 min and then maintained at 25 °C for 15 min and the FP HS bar represents cells that were treated with 500 nM flavopiridol for 40 min and then maintained at 37 °C for 15 min. Data information: In (a–d), data are presented as mean ± SEM (*n* = 2).

**Additional file 4: Figure S4.** Chromatin binding dynamics of Spt5 and the dissection of the protein–protein interaction domains of Pho. a ChIP-qPCR measurements of occupancy levels of FLAG-Spt5 at the *hsp70* locus over the time course detailed in Fig. 1a. The control line represents cells that were maintained at 25 °C for the entire duration of the time course detailed in Fig. 1a. The cartoon at the bottom of the figure represents the distance of the location of the primers used from the *hsp70* TSS. b–c co-IP assays of S2 DRSC cells transiently transfected with plasmids expressing FLAG-tagged dSfmbt or Spt5 (FLAG-dSfmbt, FLAG-Spt5) and HA-tagged Pho, N-Pho (a.a 1-351), 1-200N-Pho (a.a 1-200), 173-351N-Pho (a.a 173-351), C-Pho (a.a 352-520). Cell lysates were used for pull-downs using an anti-FLAG antibody and were later probed by Western blot using an anti-HA antibody. MW = molecular weight in kDa. d co-IP assays for S2 DRSC cells transiently transfected with plasmids expressing FLAG-tagged Pho and HA-tagged Spt5 or HA-tagged dSfmbt. S2 DRSC cells were either maintained at normal growth temperature (25 °C) or heat shocked at 37 °C for 15 min. Cell lysates were used for pull-downs using an anti-FLAG antibody and were later probed by Western blot using an anti-HA antibody. MW = molecular weight in kDa.

**Additional file 5: Figure S5.** The dynamic interaction switch between Pho and Spt5 is independent of DNA but dependent upon RNA. a co-IP assays of S2 DRSC cells transiently transfected with plasmids expressing FLAG-tagged Spt5 (FLAG-Spt5) and HA-tagged Pho (HA-Pho). S2 DRSC cells were either maintained at normal growth temperature (25 °C) or heat shocked at 37 °C for 15 min. Prior to performing the pull-down, the cell lysates were treated with either *RNase A* or *DNase I.* Thereafter, the treated cell lysates were used for pull-downs using an anti-FLAG antibody and later probed by Western blot using an anti-HA antibody. b co-IP assays of S2 DRSC cells transiently transfected with plasmids expressing FLAG-tagged Spt5 (FLAG-Spt5) and HA-tagged N-Pho (HA-N-Pho). S2 DRSC cells were either maintained at normal growth temperature (25 °C) or heat shocked at 37 °C for 15 min. Cell lysates were prepared and were used for pull-downs using an anti-FLAG antibody. They were later probed by Western blot using an anti-HA antibody.

**Additional file 6: Figure S6.** The dynamic interaction switch between Pho V164D–Spt5 is independent of DNA but dependent upon RNA. a co-IP assays of S2 DRSC cells transiently transfected with plasmids expressing FLAG-tagged Spt5 (FLAG-Spt5) and HA-tagged Pho V164D (HA-Pho V164D). S2 DRSC cells were either maintained at normal growth temperature (25 °C) or heat shocked at 37 °C for 15 min. Prior to performing the pull-down, the cell lysates were treated with either *RNase A* or *DNase I.* Thereafter, the treated cell lysates were used for pull-downs using an anti-FLAG antibody and later probed by Western blot using an anti-HA antibody. b A western blot depicting the levels of the copper-inducible constructs (Pho WT/Pho V164D) after 72 h of induction to the endogenous Pho. MW = molecular weight in kDa.

**Additional file 7: Table S1.** List of the primer sequences used in the study.

**Additional file 8: Table S2.** Amount of antibody used per IP in ChIP.

## Abbreviations

PRC1: Polycomb repressive complex 1
*hsp70*: Heat shock protein 70
HSF: Heat shock factor
Pho: Pleiohomeotic
PRE: Polycomb response element
DSIF: DRB sensitivity-inducing factor
NELF: Negative elongation factor
P-TEFb: Positive transcription elongation factor
dSfmbt: *Drosophila* Scm-related gene containing four MBT domains
Spt5: Suppressor of TY’s
CTD: C-terminal domain
S2P: Serine 2 phosphorylated
ChIP: Chromatin immunoprecipitation
N-ChIP: Native chromatin immunoprecipitation
co-IP: Co-immunoprecipitation
YY1: Ying yang 1
REPO: Recruitment of Polycomb domain
