# Supplementary figures and images for: Pho dynamically interacts with Spt5 to facilitate transcriptional switches at the hsp70 locus
Source: Epigenetics Chromatin. 2017 Dec 6;10:57. doi: 10.1186/s13072-017-0166-9 (PMC5718073; doi:10.1186/s13072-017-0166-9)

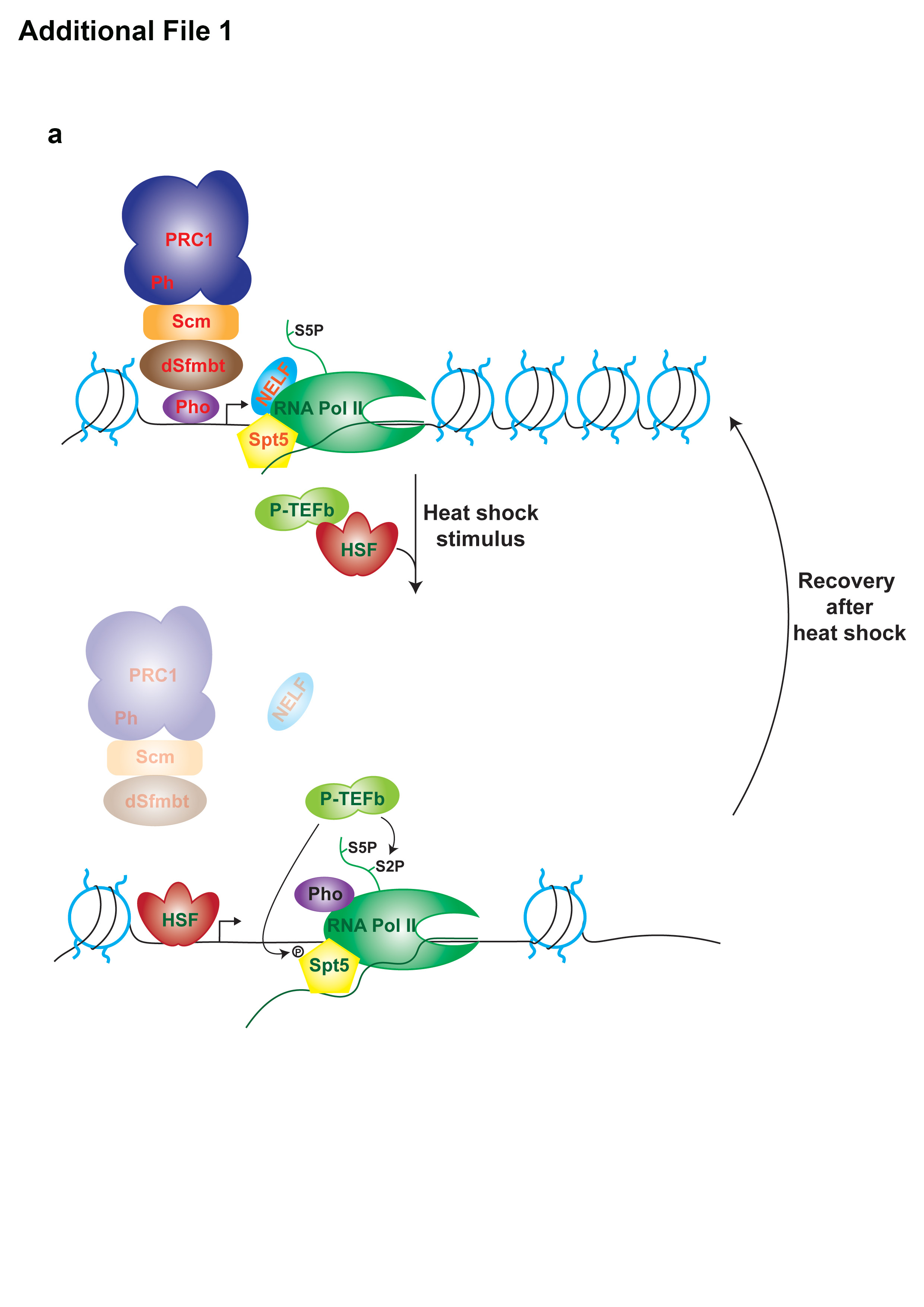

Supplement: Supplementary file 1 — Additional file 1: Figure S1. Schematic representation of the proteins involved in the silencing, activation and re-silencing of the hsp70 locus. a At optimal growth temperature, Pho, a DNA-binding PcG member, binds to promoter region of the hsp70 locus. Pho interacts with dSfmbt, which together form a recruitment platform for PRC1 to the hsp70 locus. In addition, RNA polymerase II is maintained in the paused state by NELF and Spt5, which act as pausing factors. Upon heat shock, HSF, along with P-TEFb, is recruited to chromatin and releases RNA polymerase II from the paused state. P-TEFb modifies Spt5 and converts into an elongation factor. It also modifies the CTD of RNA polymerase II to enable productive elongation. However, upon removal of the heat shock stimulus, the locus should eventually return to its paused state. Thus, hsp70 is an ideal model gene to study the eviction and recruitment of PRC1 upon activation and re-silencing, respectively. The colour code for the protein names is as follows: silencing in red, pausing factors in orange and activators in green. [file 13072_2017_166_MOESM1_ESM.jpg]

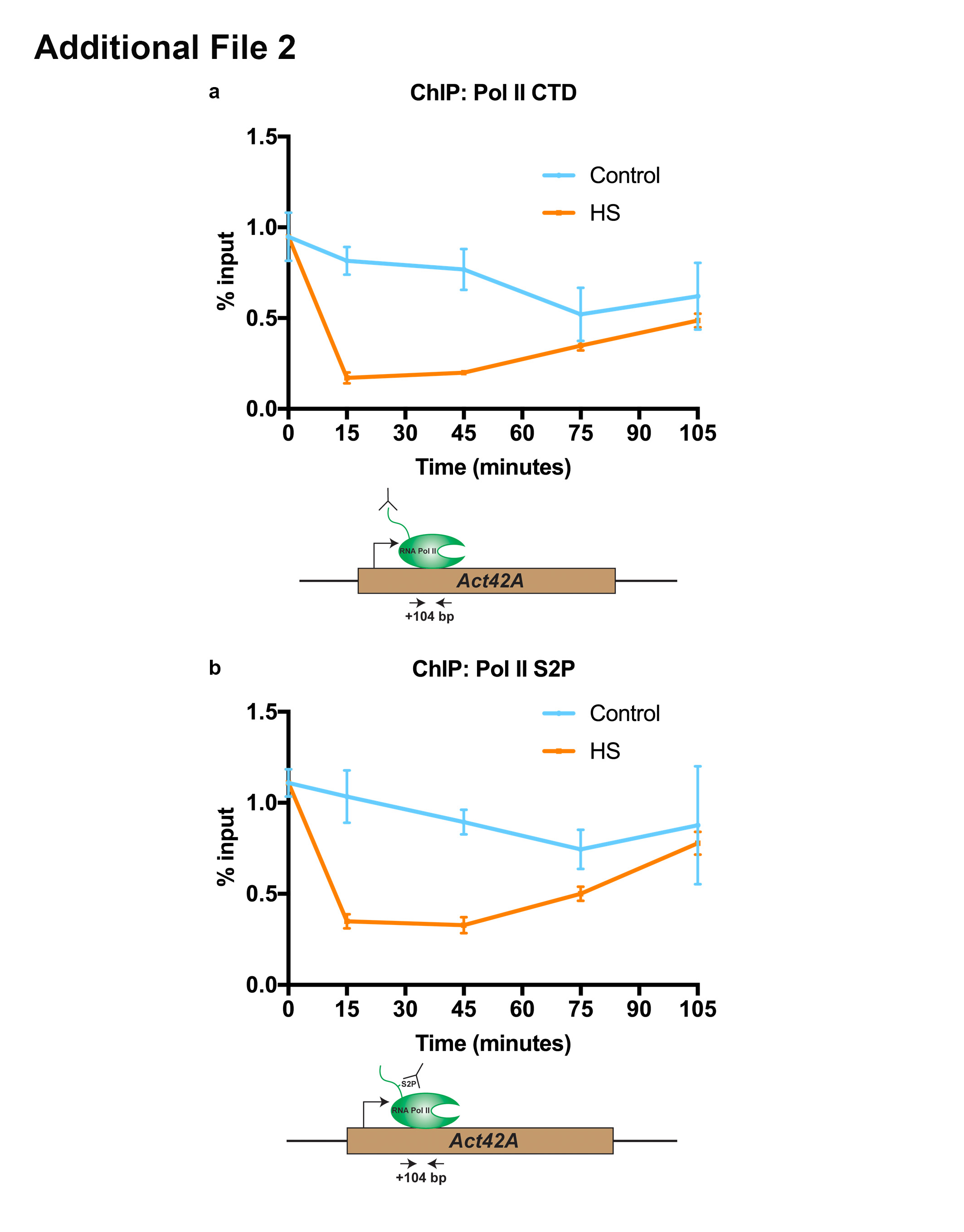

Supplement: Supplementary file 2 — Additional file 2: Figure S2. RNA polymerase II binding dynamics at Act42A locus during the heat shock response. a, b ChIP-qPCR measurements of occupancy levels of RNA polymerase II CTD and S2P form of RNA polymerase II at the Act42A locus during the heat shock response detailed in Fig. 1a. The control line represents cells that were maintained at 25 °C for the entire duration of the time course detailed in Fig. 1a. Distance of the location of the primers used from the Act42A TSS is as follows: for a and b (+ 104 bp) and has been depicted in the form of a cartoon below the data figure. Data information: In (a, b), data are presented as mean ± SEM for n = 2. [file 13072_2017_166_MOESM2_ESM.jpg]

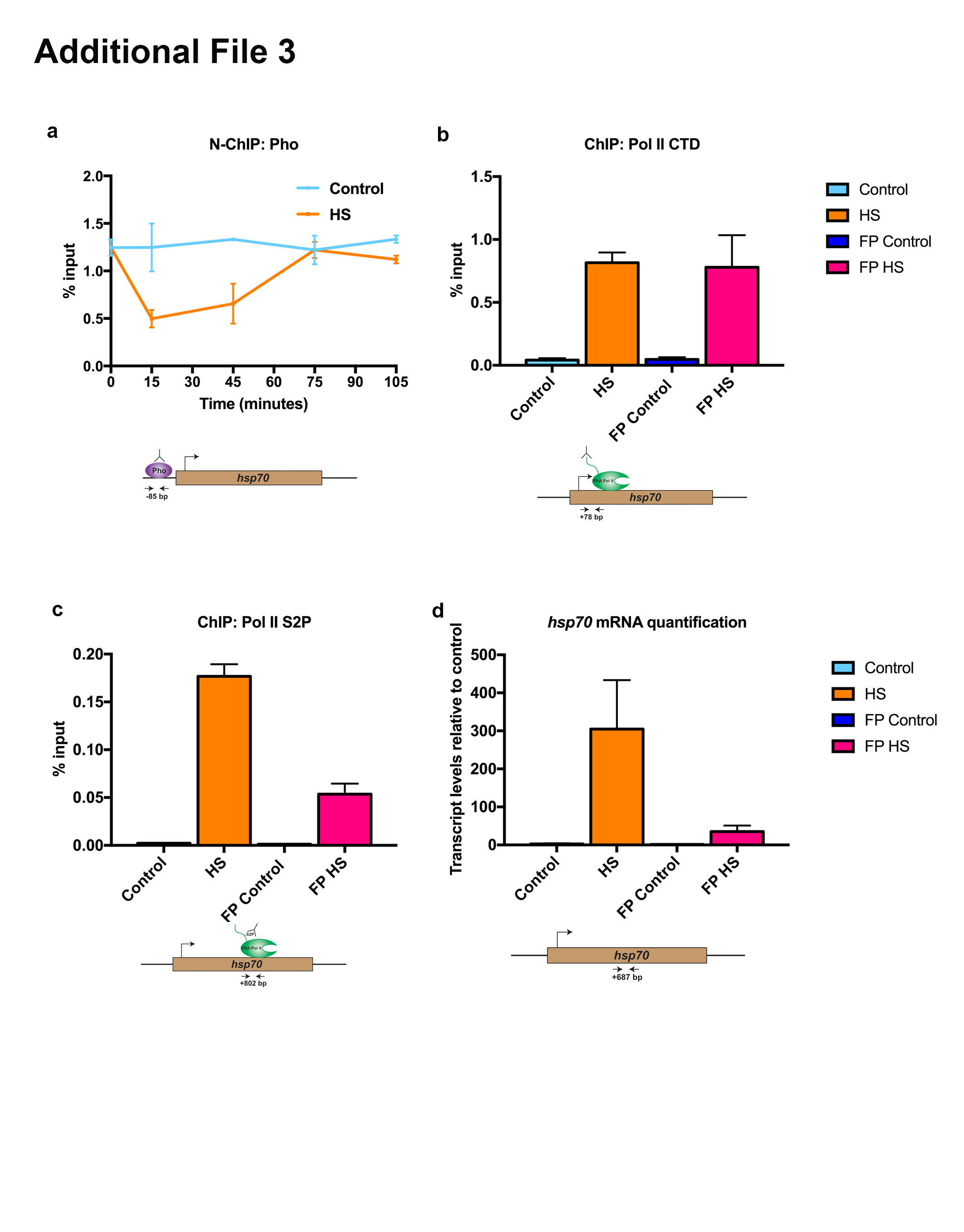

Supplement: Supplementary file 3 — Additional file 3: Figure S3. Effect of flavopiridol on RNA polymerase II occupancy at the hsp70 locus upon heat shock. a N-ChIP-qPCR measurement of the occupancy level of Pho at the hsp70 locus in S2 DRSC cells during the heat shock response detailed in Fig. 1a. Distance of the location of the primers used from the hsp70 TSS is − 85 bp and has been depicted in the form of a cartoon below the data figure. b, c ChIP-qPCR measurements of occupancy levels of RNA polymerase II CTD and S2P form of RNA polymerase II, respectively, at the hsp70 locus in S2 DRSC cells. Distances of the location of the primers used from the hsp70 TSS are as follows: for a (+ 78 bp) and for b (+ 802 bp) and has been depicted in the form of a cartoon below the data figure. d qRT-PCR measurements of hsp70 transcript levels in third-instar Drosophila larvae under the conditions used for double polytene immunostaining. Distance of the location of the primers used from the hsp70 TSS is + 687 bp and has been depicted in the form of a cartoon below the data figure. For b–d, the control bar represents cells that were maintained at 25 °C for 15 min, the HS bar represents cells that were maintained at 37 °C for 15 min, the FP control bar represents cells that were treated with 500 nM flavopiridol for 40 min and then maintained at 25 °C for 15 min and the FP HS bar represents cells that were treated with 500 nM flavopiridol for 40 min and then maintained at 37 °C for 15 min. Data information: In (a–d), data are presented as mean ± SEM (n = 2). [file 13072_2017_166_MOESM3_ESM.jpg]

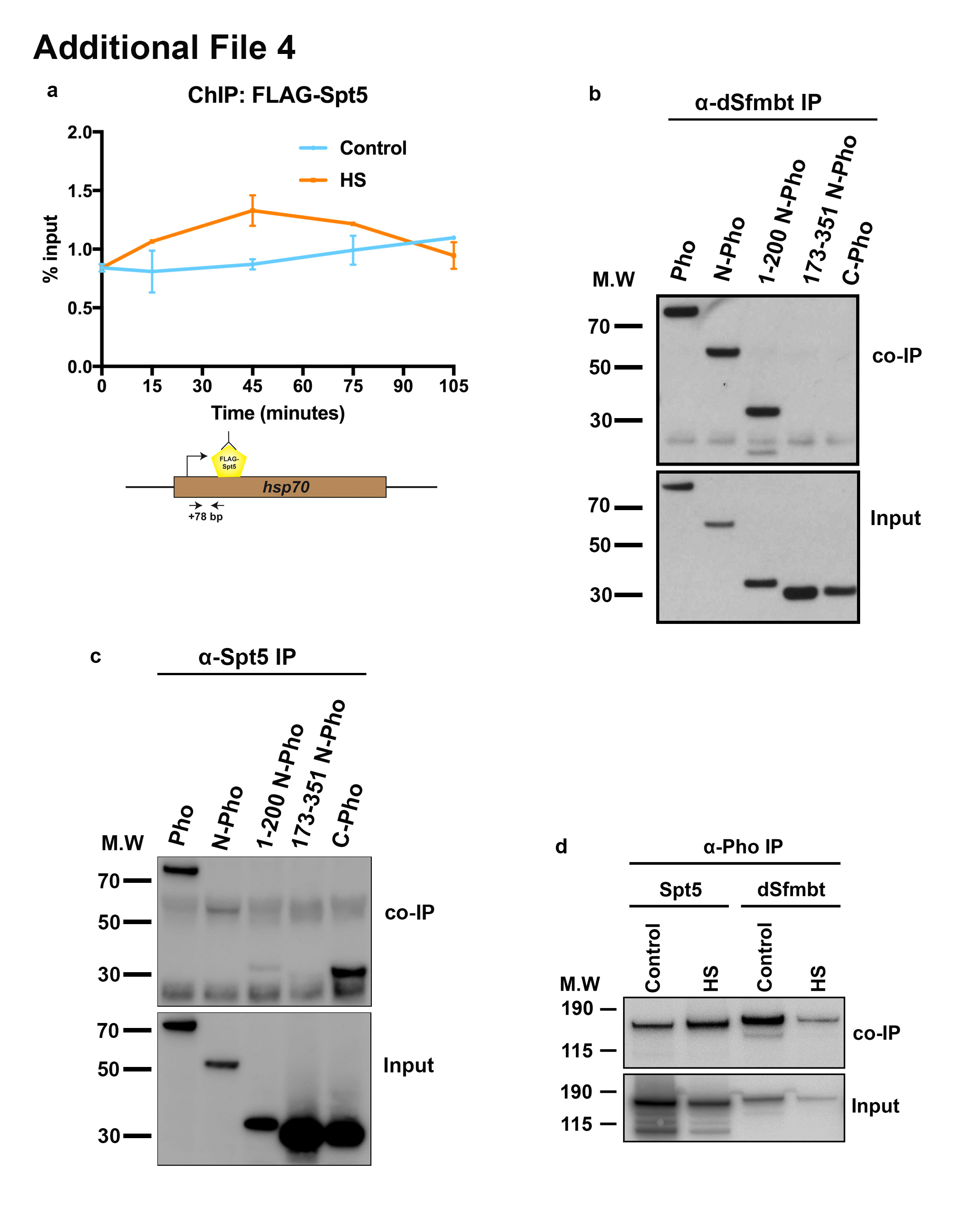

Supplement: Supplementary file 4 — Additional file 4: Figure S4. Chromatin binding dynamics of Spt5 and the dissection of the protein–protein interaction domains of Pho. a ChIP-qPCR measurements of occupancy levels of FLAG-Spt5 at the hsp70 locus over the time course detailed in Fig. 1a. The control line represents cells that were maintained at 25 °C for the entire duration of the time course detailed in Fig. 1a. The cartoon at the bottom of the figure represents the distance of the location of the primers used from the hsp70 TSS. b–c co-IP assays of S2 DRSC cells transiently transfected with plasmids expressing FLAG-tagged dSfmbt or Spt5 (FLAG-dSfmbt, FLAG-Spt5) and HA-tagged Pho, N-Pho (a.a 1-351), 1-200N-Pho (a.a 1-200), 173-351N-Pho (a.a 173-351), C-Pho (a.a 352-520). Cell lysates were used for pull-downs using an anti-FLAG antibody and were later probed by Western blot using an anti-HA antibody. MW = molecular weight in kDa. d co-IP assays for S2 DRSC cells transiently transfected with plasmids expressing FLAG-tagged Pho and HA-tagged Spt5 or HA-tagged dSfmbt. S2 DRSC cells were either maintained at normal growth temperature (25 °C) or heat shocked at 37 °C for 15 min. Cell lysates were used for pull-downs using an anti-FLAG antibody and were later probed by Western blot using an anti-HA antibody. MW = molecular weight in kDa. [file 13072_2017_166_MOESM4_ESM.jpg]

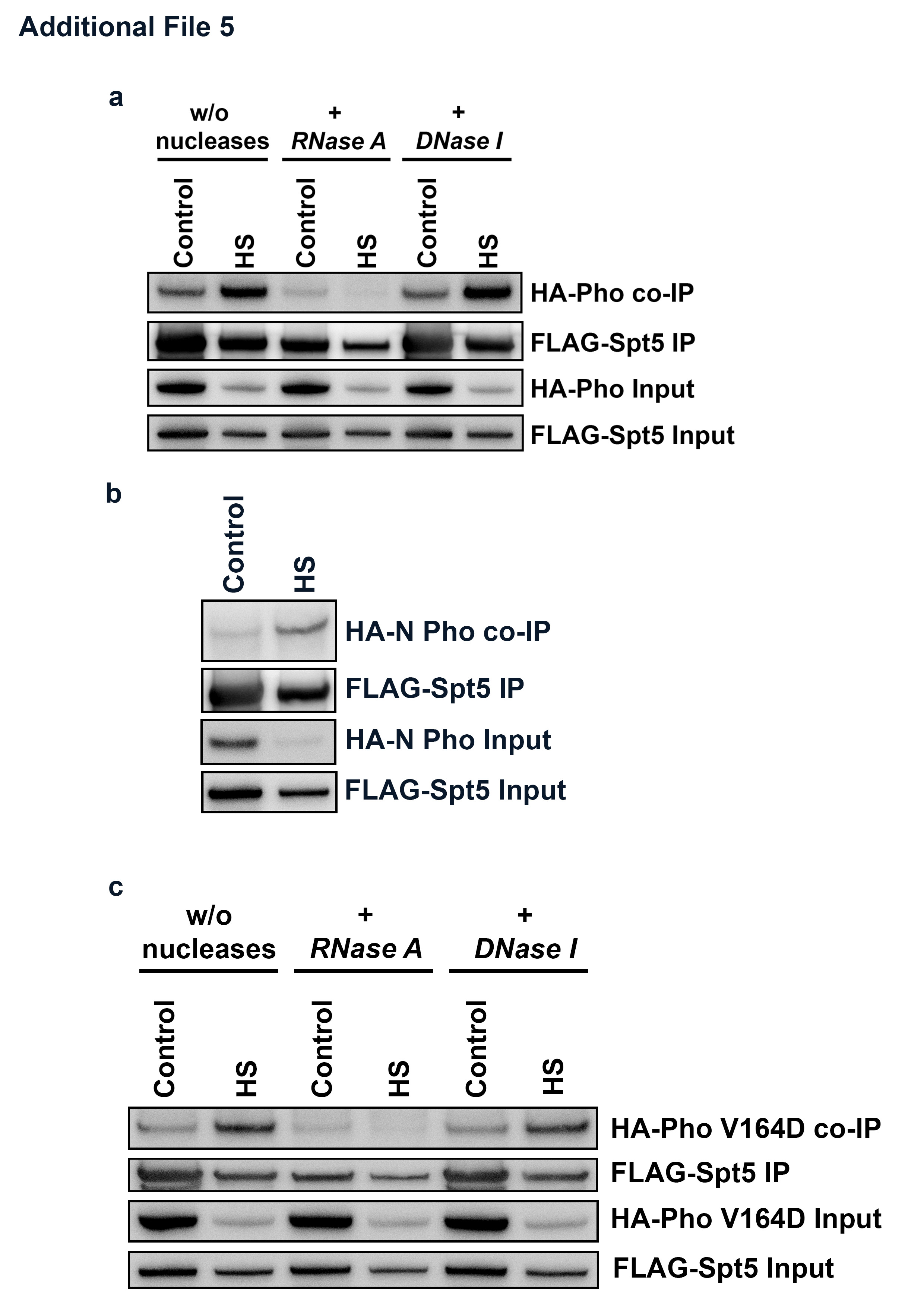

Supplement: Supplementary file 5 — Additional file 5: Figure S5. The dynamic interaction switch between Pho and Spt5 is independent of DNA but dependent upon RNA. a co-IP assays of S2 DRSC cells transiently transfected with plasmids expressing FLAG-tagged Spt5 (FLAG-Spt5) and HA-tagged Pho (HA-Pho). S2 DRSC cells were either maintained at normal growth temperature (25 °C) or heat shocked at 37 °C for 15 min. Prior to performing the pull-down, the cell lysates were treated with either RNase A or DNase I. Thereafter, the treated cell lysates were used for pull-downs using an anti-FLAG antibody and later probed by Western blot using an anti-HA antibody. b co-IP assays of S2 DRSC cells transiently transfected with plasmids expressing FLAG-tagged Spt5 (FLAG-Spt5) and HA-tagged N-Pho (HA-N-Pho). S2 DRSC cells were either maintained at normal growth temperature (25 °C) or heat shocked at 37 °C for 15 min. Cell lysates were prepared and were used for pull-downs using an anti-FLAG antibody. They were later probed by Western blot using an anti-HA antibody. [file 13072_2017_166_MOESM5_ESM.jpg]

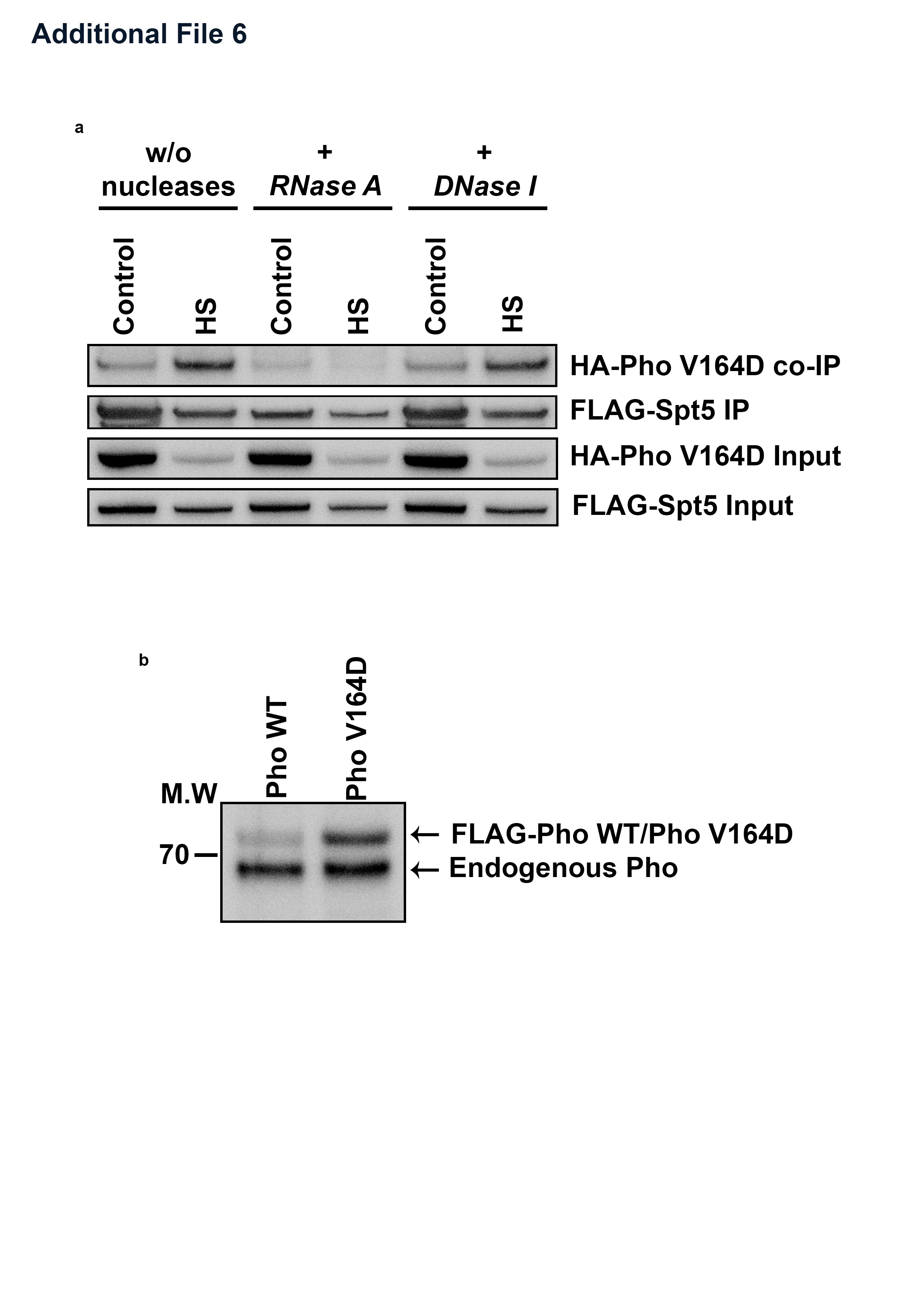

Supplement: Supplementary file 6 — Additional file 6: Figure S6. The dynamic interaction switch between Pho V164D–Spt5 is independent of DNA but dependent upon RNA. a co-IP assays of S2 DRSC cells transiently transfected with plasmids expressing FLAG-tagged Spt5 (FLAG-Spt5) and HA-tagged Pho V164D (HA-Pho V164D). S2 DRSC cells were either maintained at normal growth temperature (25 °C) or heat shocked at 37 °C for 15 min. Prior to performing the pull-down, the cell lysates were treated with either RNase A or DNase I. Thereafter, the treated cell lysates were used for pull-downs using an anti-FLAG antibody and later probed by Western blot using an anti-HA antibody. b A western blot depicting the levels of the copper-inducible constructs (Pho WT/Pho V164D) after 72 h of induction to the endogenous Pho. MW = molecular weight in kDa. [file 13072_2017_166_MOESM6_ESM.jpg]
